# A switch element in the autophagy E2 Atg3 mediates allosteric regulation across the lipidation cascade

**DOI:** 10.1038/s41467-019-11435-y

**Published:** 2019-08-09

**Authors:** Yumei Zheng, Yu Qiu, Christy R. R. Grace, Xu Liu, Daniel J. Klionsky, Brenda A. Schulman

**Affiliations:** 1 Department of Structural Biology, St. Jude Children’s Research Hospital, Memphis, TN USA; 2 Department of Microbiology, Immunology and Biochemistry, University of Tennessee Health Science Center, Memphis, TN USA; 3 Life Sciences Institute, University of Michigan, Ann Arbor, MI USA; 4 Department of Molecular Machines and Signaling, Max Planck Institute of Biochemistry, Martinsried, Germany; 5 Present Address: Biologics Research, Sanofi, Framingham, MA USA; 6 Division of Infectious Diseases, Brigham and Women’s Hospital, Boston, MA USA; 7 Department of Microbiology and Immunobiology, Harvard Medical School, Boston, MA USA

**Keywords:** Solution-state NMR, Ligases, Enzyme mechanisms, Post-translational modifications

## Abstract

Autophagy depends on the E2 enzyme, Atg3, functioning in a conserved E1-E2-E3 trienzyme cascade that catalyzes lipidation of Atg8-family ubiquitin-like proteins (UBLs). Molecular mechanisms underlying Atg8 lipidation remain poorly understood despite association of Atg3, the E1 Atg7, and the composite E3 Atg12–Atg5-Atg16 with pathologies including cancers, infections and neurodegeneration. Here, studying yeast enzymes, we report that an Atg3 element we term E123IR (E1, E2, and E3-interacting region) is an allosteric switch. NMR, biochemical, crystallographic and genetic data collectively indicate that in the absence of the enzymatic cascade, the Atg3^E123IR^ makes intramolecular interactions restraining Atg3′s catalytic loop, while E1 and E3 enzymes directly remove this brace to conformationally activate Atg3 and elicit Atg8 lipidation in vitro and in vivo. We propose that Atg3′s E123IR protects the E2~UBL thioester bond from wayward reactivity toward errant nucleophiles, while Atg8 lipidation cascade enzymes induce E2 active site remodeling through an unprecedented mechanism to drive autophagy.

## Abstract (web summary)

Autophagy mediated by the conjugation pathway for ubiquitin-like proteins plays a key role in controlling homeostasis in eukaryotic cells. Here the authors provide a molecular basis for allosteric activation of the E2 ligase Atg3, uncovering the mechanism underlying Atg8 lipidation and a novel mechanism regulating E1-E2-E3-mediated ubiquitin-like protein conjugation.

## Introduction

Autophagy is a major catabolic pathway by which eukaryotic cells degrade and recycle diverse cellular materials^1^. Sectors of the cytosol are first sequestered within a double-membrane-bound autophagosome, and then degraded upon autophagosome fusion with the lysosome in higher eukaryotes or vacuole in yeast^2^. Cargoes directed to autophagosomes for degradation, include protein assemblies, organelles, misfolded macromolecules, debris, and pathogenic bacteria^3–5^. As such, autophagy plays important roles in cellular metabolism in many physiological processes, while dysregulation of autophagy is associated with metabolic, neurologic, and oncologic disorders^6–12^.

Many facets of autophagy rely on the autophagy-specific ubiquitin-like protein (UBL) Atg8 in yeast (or six LC3- and GABARAP-family UBLs in higher eukaryotes, here referred to collectively as Atg8-family members) and the E1–E2–E3 enzymatic cascade that links Atg8′s C-terminus to the primary amino head group of phosphatidylethanolamine (PE) lipid molecules^13–16^. Ultimately, lipidated Atg8-family members serve as bridges between membranes and the cytosol, where their ubiquitin-like fold domains recruit Atg8-interacting motif (AIM) or LC3-interaction region (LIR) sequences in a staggering array of partner proteins involved in various aspects of autophagy^17–20^. These, include regulators and effectors of autophagosome biogenesis that promote membrane tethering and growth of the autophagosome, as well as adaptor proteins that in turn recruit cargoes for degradation^21^. Accordingly, Atg8 and its conjugation system enzymes are essential for autophagy in yeasts, and autophagy is severely impaired in their absence in mammalian cells^22^. Lipidated Atg8-family members have also been implicated in protein recruitment to membranes in processes that are unrelated to autophagy in mammalian cells^23^. Therefore, given these important roles of Atg8 and its conjugation system enzymes, it is important to understand molecular mechanisms regulating Atg8 lipidation.

The central enzyme in the Atg8 lipidation cascade is the E2, Atg3^13,24^. Atg3 receives Atg8 from the E1 Atg7, to form a covalent, reactive Atg3~Atg8 intermediate linked by a thioester bond (referred to by ~) between the C-terminus of Atg8 and the catalytic Cys of Atg3. Atg8 is then transferred from the Atg3 catalytic Cys to PE in a reaction catalyzed by a composite E3 enzyme^25^ containing another autophagy UBL, Atg12, conjugated to Atg5^26^. In vitro, the yeast Atg12–Atg5 conjugate is sufficient to serve as an E3 enzyme activating lipidation of Atg8^26^. Atg16 binding to lipids substantially enhances E3 activity^27^ and is required in vivo^28^ to localize Atg12–Atg5 to the preautophagosomal structure that precedes autophagosome formation^29,30^, through many mechanisms^31–33^.

**Fig. 1:**
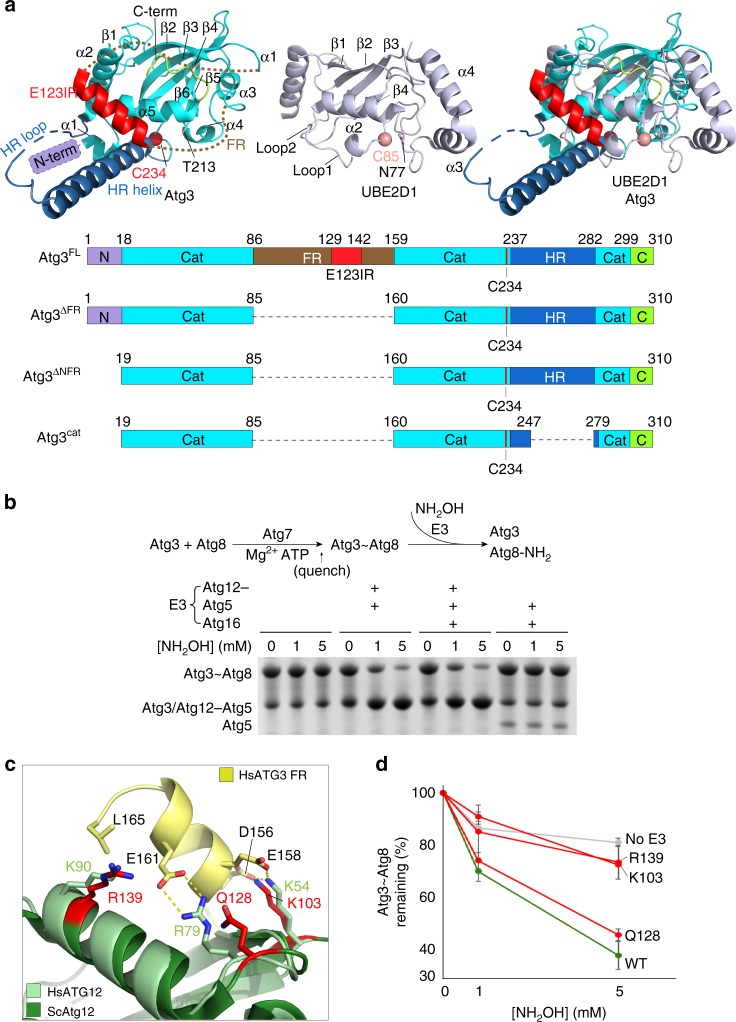
The Atg12–Atg5 module within the autophagy E3 activates intrinsic reactivity of the Atg3~Atg8 intermediate. **a** Structures of yeast Atg3 (left, PDB 2DYT), a ubiquitin E2 (middle, UBE2D1, PDB 4AP4), and their superimposition (right). Atg3 unique elements are indicated by colors shown in schematics of Atg3 constructs used in this study (below). **b** Intrinsic reactivity of Atg3~Atg8 intermediate monitored by pulse-chase discharge to NH_2_OH in presence of indicated E3 variant. **c** Close-up of structural superimposition of yeast Atg12–Atg5-Atg16 (PDB 3W1S) and corresponding human complex with ATG3^FR^ (PDB 4NAW). Residues from yeast Atg12 corresponding to the human ATG3^FR^-binding site are shown as red sticks. **d** Effects of mutations in minimal yeast E3 (Atg12–Atg5) on stimulating Atg3~Atg8 discharge, monitored by quantification of Atg3~Atg8 remaining after 2.5 min in pulse-chase assays using the scheme as in (**b**), as a function of NH_2_OH concentration. (Error bar: STDEV, *N* = 3) Representative gel is shown in Supplemental Fig. 1b; all points are shown in Source Data File

The prior structure of Atg3 from *S. cerevisiae* revealed a catalytic core domain (Atg3^cat^) largely resembling E2s for canonical UBLs such as Ub, NEDD8 and SUMO, including a long central helix and two catalytic segments^34^ (Fig. 1a). One segment harbors the catalytic Cys234, and the other displays Thr213, which also is essential for the lipidation reaction and corresponds to the canonical E2 catalytic Asn that plays a crucial structural role^13,35,36^. In addition, Atg3 displays unique, functionally important structures^34,37,38^: an N-terminal amphipathic membrane-binding helix (N, residues 1–18); an intrinsically disordered flexible region (FR, residues 85–159); a handle region consisting of a helix and a disordered region (HR, residues 237–282) that contains an Atg8-interacting motif that is dispensable for the lipidation reaction but plays important roles in vivo; and a flexible extreme C-terminal extension (C, residues 299–310). While prior structural studies showed how Atg7 engages Atg3^cat^ and the Atg3^FR^ for Atg8 transfer to Atg3^39–43^, less is understood about subsequent roles of the autophagy E3. A crystal structure showed how the human E3 enzyme recruits a peptide-like region from Atg3′s FR, but this FR motif is not conserved across Atg3 sequences from many organisms, including yeasts^44^. Furthermore, the structure lacked Atg3′s catalytic domain, for which prior studies of cysteine accessibility in the yeast enzyme suggested that the autophagy E3 triggers a structural rearrangement that affects the orientation of the catalytic Cys234 relative to Thr213^35^. The molecular underpinnings for E3-dependent conformational activation remains elusive.

Here, we use a hybrid structural approach integrating information from biochemistry, nuclear magnetic resonance (NMR), crystallography, and yeast genetics to identify a molecular basis for Atg3 allosteric activation. Overall, the data suggest that a distinct peptide-like element acts like a brace mediating intramolecular interactions with the Atg3 catalytic domain to restrict the conformation of the active site and prevent reactivity toward errant nucleophiles, while the E3 elicits an unprecedented E2 activation mechanism by remotely binding to this brace to couple other E3 interactions with allosteric activation of active site mediating Atg8 lipidation.

## Results

### Autophagy E3 activates intrinsic reactivity of Atg3~Atg8

E2–UB and E2–UBL intermediates are activated in distinct ways by different classes of E3 enzymes^45–47^. Many E3s, for example RING and related E3s for UB, NEDD8 and SUMO, and SIM-containing SUMO E3s, allosterically promote noncovalent interactions between the E2 and UB (or UBL) moieties within the covalent intermediate. This is often monitored by an E3 increasing the susceptibility of an E2–UB or E2–UBL intermediate to nucleophilic attack by a nonspecific amine nucleophile^48^. In contrast, HECT and RBR E3s use a different mechanism that depends on positioning the E2-bound UB for transfer to the E3 catalytic Cys. Although the autophagy E3 lacks homology to UB, NEDD8, and SUMO ligases, we asked whether there are common mechanistic features by testing biochemical activities of the relatively simplistic Atg8 pathway from *S. cerevisiae*. Potential E3-mediated stimulation of intrinsic activity of the Atg3~Atg8 intermediate was examined by adapting the pulse-chase assay format used to study canonical UB enzymes (Fig. 1b). Briefly, a pulse-reaction catalyzed by Atg7 generated the thioester-linked Atg3~Atg8 intermediate. After quenching this reaction with apyrase, increasing concentrations of hydroxylamine were added (Fig. 1b). Discharge of the Atg3~Atg8 intermediate to this nonspecific amine nucleophile was monitored by appearance of free Atg3 in sodium dodecyl sulphate polyacrylamide gel electrophoresis (SDS-PAGE). Whereas the Atg3~Atg8 intermediate was relatively stable on its own, addition of Atg12–Atg5 stimulated discharge. This activation was maintained in the presence of Atg16, while Atg5-Atg16 alone was insufficient (Fig. 1b). Atg12–Atg5 is also known to be required for E3 activation of the lipidation reaction^26,27,35^. Thus, within the composite autophagy E3 is a module that activates the intrinsic reactivity of the Atg3~Atg8 thioester-linkage.

As a first step toward understanding the mechanism, we performed mutagenesis based on the prior structure showing that in the context of the human autophagy E3, Atg12 recruits a short peptide-like region of Atg3′s FR^44^. Although the FR sequences from human and yeast Atg3 are not conserved (Supplementary Fig. 1a), the structures of human and yeast Atg12–Atg5 superimpose well, including the Atg3^FR^-binding site (Fig. 1c)^49,50^. Mutations in the corresponding surface of yeast Atg12–Atg5 not only impair Atg8 lipidation but also intrinsic activation of the Atg3~Atg8 intermediate (Fig. 1d and Supplementary Fig. 1b, c). Thus, it seems probable that the FR from yeast Atg3 likewise contains an E3-binding element.

### Identification of an E3-binding element in yeast Atg3

**Fig. 2:**
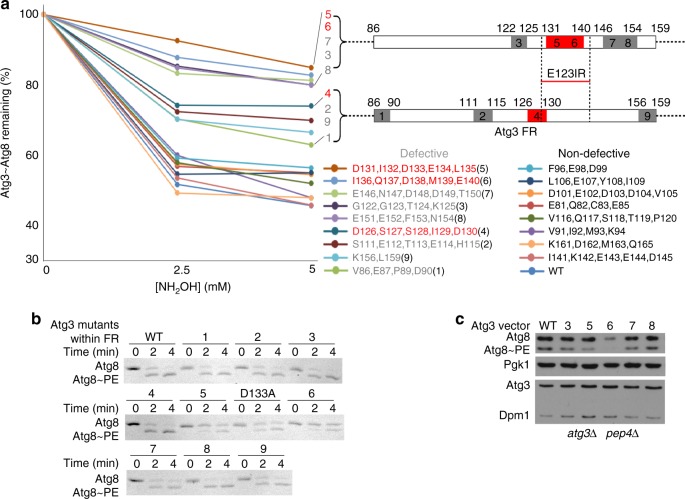
E123IR plays essential role in E3-dependent Atg8 ligation activity. **a** Effects of indicated Ala mutants in Atg3′s FR on E3 (Atg12–Atg5-Atg16) activation of Atg3~Atg8 intermediate, quantified as percent Atg3~Atg8 remaining in pulse-chase discharge to NH_2_OH over 2.5 min. Locations of most defective and moderately defective mutants are indicated on schematics, and those corresponding to Atg3^E123IR^ are shown in red. **b** Effects of indicated Ala mutants in Atg3′s FR on Atg8 lipidation in vitro, in reactions with Atg7, Atg12–Atg5-Atg16, and liposomes generated from *E. coli* polar lipids as source of PE, and detected by migration of Atg8 in Coommassie-stained SDS-PAGE gel. **c** Effects of indicated Ala mutants in Atg3′s FR on Atg8 lipidation in vivo, as detected by western blot for Atg8 after 2 h starvation of XLY161 *atg3∆pep4∆* strain of *S. cerevisiae* expressing either WT or mutant HA-tagged Atg3. Pgk1 is loading control for Atg8, Dpm1 is loading control for Atg3

**Fig. 3:**
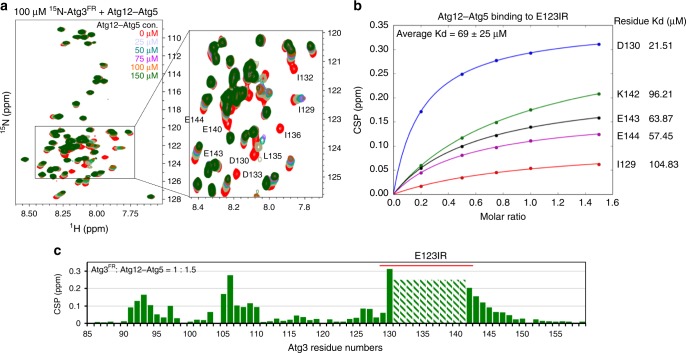
E123IR interacts with autophagy E3. **a** [^15^N, ^1^H] TROSY spectra of ^15^N-labeled Atg3^FR^ titrated with unlabeled Atg12–Atg5-Atg16. **b** Estimated binding affinities between E123IR residues and Atg12–Atg5, based on chemical shift perturbations (CSPs) observed upon titrating increasing concentrations of Atg12–Atg5. **c** Chemical shift perturbations plotted as a function of Atg3^FR^ residue numbers, with resonances showing intermediate exchange line broadening indicated by stripes

A multi-tiered approach was used to identify the Atg3 element determining E3 activation and binding. First, given that the corresponding E3-binding element from human Atg3 is located in the FR, a panel of Atg3 variants harboring individual or multiple alanine mutations in the FR were monitored for E3-dependent Atg3~Atg8 discharge (Fig. 2a and Supplementary Fig. 2). Second, mutants hindering discharge were tested for relevance to autophagy, by testing for effects on E3-dependent Atg8 lipidation in vitro and in vivo (Fig. 2b, c). Finally, NMR was used to define the Atg3^FR^ region mediating E3-binding. After resonances were assigned for a construct spanning the entire intrinsically disordered Atg3^FR^ (residues 86–159), an unlabeled version of the Atg12–Atg5 conjugate was added, and [^15^N, ^1^H] TROSY spectra were examined for chemical shift perturbation and/or line-broadening as an indicator of binding (Fig. 3a, c). Although other residues in Atg3′s FR contribute to activity (Fig. 2a), the major region implicated by all assays spans from Ile129-Lys142 (Fig. 2a–c and Fig. 3a, c). For reasons described below, we termed this region Atg3^E123IR^.

### Atg3′s E3-binding element also binds E1 and E2

**Fig. 4:**
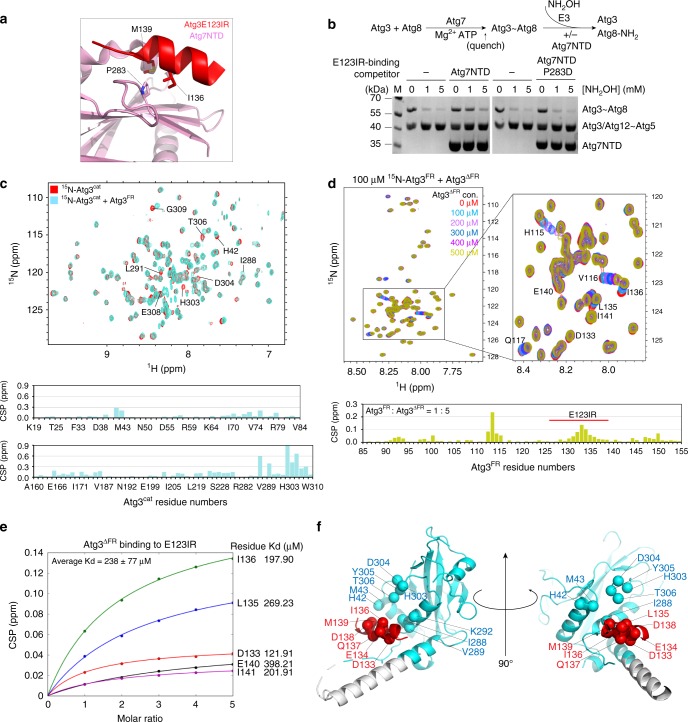
E123IR binds the E1 Atg7 and E2 core domain Atg3^cat^. **a** Close-up showing interactions between Atg3^E123IR^ and N-terminal domain (NTD) of Atg7 (PDB 3T7G). **b** Effects of Atg3^E123IR^-binding WT Atg7 NTD and non-binding mutant (P283D) on E3-stimulated intrinsic reactivity of Atg3~Atg8 intermediate, as monitored by pulse-chase discharge to NH_2_OH. **c** [^15^N, ^1^H] TROSY spectra of ^15^N-labeled Atg3^cat^ alone (red) or in 1:44 mixture with unlabeled Atg3^FR^ (cyan), with chemical shift perturbations (CSPs) plotted per residue below. **d** [^15^N, ^1^H] TROSY spectra of ^15^N-labeled Atg3^FR^ titrated with unlabeled Atg3^∆FR^, with representative (1:5 molar ratio Atg3^FR^ vs. Atg3^∆FR^) CSPs plotted per residue below. **e** Estimated binding affinities between E123IR residues and Atg3^∆FR^, based on CSPs observed upon titrating increasing concentrations of Atg3^∆FR^. **f** Residues corresponding to greatest CSPs are shown as spheres on the structure of Atg3 (PDB 2DYT), except E308 and G309 that are not visible in the structure

Prior studies showed that the region corresponding to Atg3^E123IR^ mediates Atg3 recruitment to the autophagy E1, Atg7^42,51^. In this upstream step of the conjugation cascade, Atg3^E123IR^ binding to Atg7′s N-terminal domain (Atg7^NTD^) anchors the complex, thereby increasing the local concentration for distal, transient interactions between Atg7′s and Atg3′s catalytic domains for Atg8 transfer between them^43^ (Fig. 4a). To further confirm that as in the human autophagy pathway^52,53^, E1 and E3 binding to Atg3 is mutually exclusive for yeast enzymes, we examined the ability of the isolated NTD from Atg7 to compete with Atg12–Atg5-dependent activation of Atg3~Atg8. The wild-type Atg7^NTD^ inhibits the E3-dependent reaction, whereas a version harboring a mutation (P283D) previously shown to impair Atg3 binding (Fig. 4a)^42^ does not show this inhibitory effect, consistent with mutually exclusive binding (Fig. 4b).

Intriguingly, this same E1- and E3-binding Atg3^E123IR^ element was also found to contact the Atg3^cat^ domain in the prior crystal structure of full-length Atg3 alone, although the interactions were previously attributable to crystal packing and not tested further (Fig. 1a)^34^. To address the potential for bona fide interaction in solution, we wished to perform NMR. This first required generation of suitable fragments from Atg3, because in prior studies of the full-length protein, it was not possible to detect resonances from the catalytic domain due to dramatically different dynamic properties from the FR region^34^. Thus, the isolated Atg3^cat^ domain was prepared (construct listed in Fig. 1a), and resonances observed in [^15^N–^1^H] TROSY spectra were assigned (Fig. 4c). Intriguingly, resonances corresponding to the active site loop (residues 232–236) were not assignable in the spectrum, potentially due to motions on the millisecond or intermediate exchange time scales.

[^15^N–^1^H] TROSY spectra obtained upon adding a synthetic peptide corresponding to the Atg3^FR^ element to ^15^N-labeled Atg3^cat^ (Fig. 4c), or upon adding an unlabeled version of Atg3^∆FR^ to ^15^N-labeled Atg3^FR^ (Fig. 4d), showed substantial chemical shift perturbations. In both titrations, the perturbed resonances correspond to the Atg3^FR^ and Atg3^cat^ domain residues observed to interact in the crystal structure (Fig. 4f). Consistent with the interactions, Introduction of mutations to hydrophobic residues I132, L135, and I136 significantly diminished the chemical shift perturbations within E123IR (Supplementary Fig. 3a), and further lower the E123IR-Atg3^∆FR^ binding affinity (Supplementary Fig. 3b). Thus, the NMR data confirmed the Atg3^E123IR^ element as a bona fide Atg3^cat^-binding element in solution. The term Atg3^E123IR^ reflects this being an **E1**-, E**2**-, and **E3**-interacting **r**egion.

Compared to the interaction with Atg12–Atg5 (Fig. 3b), we observed lower binding affinity between E123IR and Atg3^∆FR^ (Fig. 4e). To gain insights into the structure of E123IR in its free form, we examined the ΔδC^α^ − ΔδC^β^ secondary chemical shifts, which show this element is not a helix in isolation (Supplementary Fig. 3c)^54^. It seems likely that a combination of different affinities for different partners, and intrinsic conformational plasticity could enable switching between E1-, E2-, and E3-bound states.

### Active site loop conformation in absence of Atg3^E123IR^

**Fig. 5:**
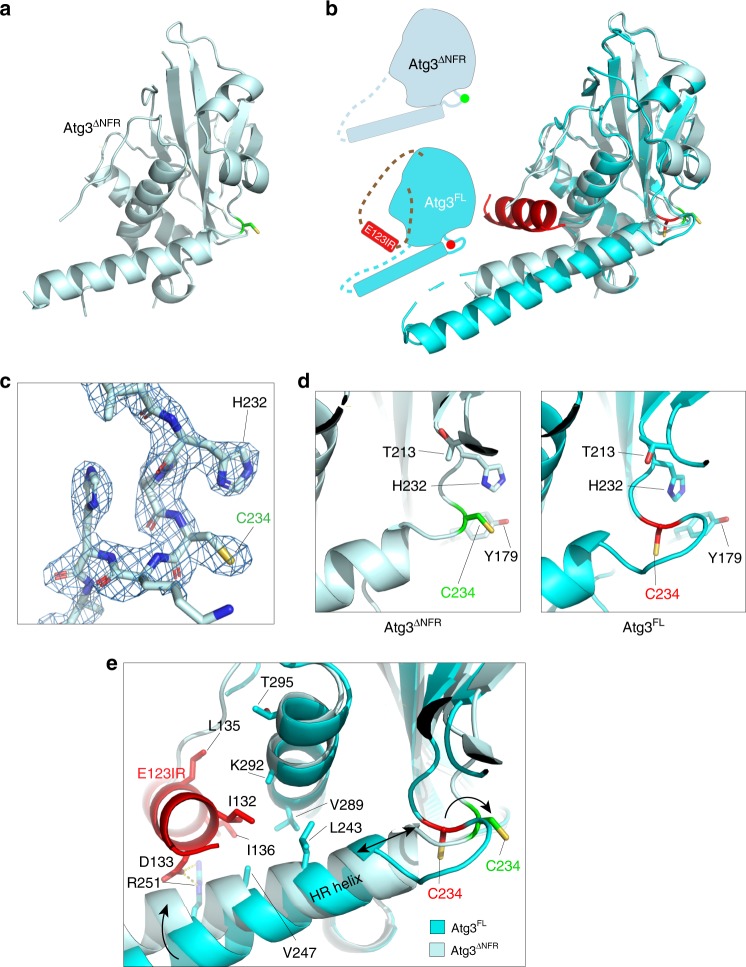
Conformational changes upon E123IR removal from Atg3. **a** Crystal structure of Atg3^∆NFR^ (PDB 6OJJ, from this study). **b** Superposition of Atg3^∆NFR^ (PDB 6OJJ, light blue with catalytic Cys shown in green) with prior structure of Atg3^FL^ (cyan, PDB 2DYT) with E123IR and catalytic Cys shown in red, and differences highlighted in cartoons. **c** Fo–Fc map shown at 3*σ* after omitting the catalytic cysteine region (residues 231–237) of Atg3^∆NFR^ and performing simulated annealing. **d** Close-ups of catalytic elements from Atg3^∆NFR^ and Atg3^FL^. **e** Close-up superposition of Atg3^∆NFR^ (light blue) and Atg3^FL^ (cyan) structures, showing interactions between Atg3′s catalytic domain and E123IR, and conformational rearrangements upon E123IR dislocation

To illuminate roles of Atg3^E123IR^-binding to the Atg3^cat^, we determined a crystal structure lacking these interactions for comparison to the prior structure of full-length Atg3. The structure of a version of Atg3 lacking the N-terminal 18 residues and the FR region (Atg3^∆NFR^) (Fig. 5a, Supplementary Fig. 4a, and Supplementary Table 1), determined at 2.5 Å resolution, superimposes well overall with that of full-length Atg3 (0.48 root-mean-square deviation over C-alphas) (Fig. 5b). However, there are substantial differences in the catalytic Cys loop and adjacent regions. As described previously, in the structure of full-length Atg3, the catalytic Cys234 is sequestered in a pocket formed by side chains from the HR and two loops^34^. This orientation is catalytically incompetent, with the Cys distal from the structurally important Thr213 and from two other side chains (Tyr179 and His232) that are important for the lipidation reaction (Fig. 5d, right)^34,35,43^. By contrast, in the absence of the Atg3^E123IR^ element, Atg3′s catalytic center is structurally rearranged into an activated conformation through a domino-like effect that ultimately results in remodeling a loop observed in full-length Atg3 into an additional helical turn at the N-terminus of the HR (Fig. 5e); the remodeled helix cannot restrain the side-chain from catalytic Cys234, thereby allowing the alternative orientation where Cys234 is surrounded by additional key catalytic residues (Fig. 5d, left). Moreover, it appears that the extreme C-terminal region of Atg3 also rearranges between the E123IR-bound and other states: this element undergoes a conformational change and additional C-terminal residues are visible in the crystal structure of Atg3^∆NFR^ relative to the prior structure of full-length Atg3. In the conformation in Atg3^∆NFR^ these residues would clash with the bound E123IR (Supplementary Fig. 4b, c), although their nearby location also suggests potential to positively interact with the E123IR in an alternative, presently unknown conformation. Indeed, resonances from these residues display large NMR chemical shift perturbations when ^15^N-labeled Atg3^cat^ is titrated with the unlabeled FR (Fig. 4c).

### Mutants displacing Atg3^E123IR^ from Atg3^cat^ activate ligation

**Fig. 6:**
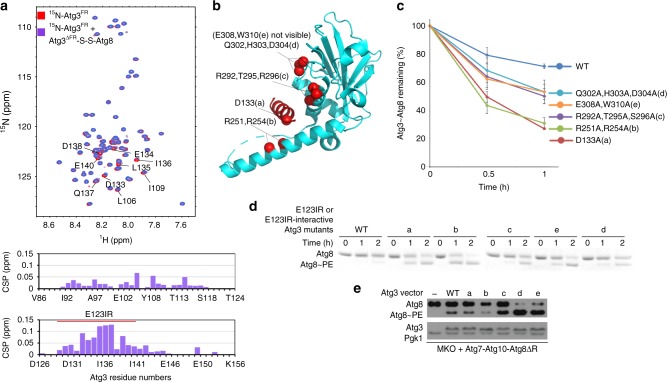
Mutations in E123IR-binding residues activate Atg3~Atg8 in the absence of E3 in vitro and in vivo. **a** [^15^N, ^1^H] TROSY spectra of ^15^N-labeled Atg3^FR^ alone (red) or in 1:2 mixture with unlabeled, disulfide-bonded proxy for Atg3^∆FR^–Atg8 (purple), with chemical shift perturbations (CSPs) per residue shown below. **b** Locations of mutations **a**–**e** designed to impair interactions between Atg3′s catalytic domain and E123IR shown on crystal structure of Atg3 (PDB 2DYT). **c** Effects of indicated mutants in Atg3 catalytic domain-E123IR interface on intrinsic E3-independent activity of Atg3~Atg8 intermediate. Quantification is of WT or indicated mutant versions of Atg3~Atg8 remaining after 2.5 min as a function of NH_2_OH concentration in pulse-chase assays, without E3, repeated 3 times repeats average (Error bar: STDEV, *N* = 3). Representative gel is shown in Supplemental Fig. 5; all data points are shown in Source Data file. **d** Effects of indicated mutants in Atg3 catalytic domain-E123IR interface on E3-independent Atg8 lipidation in vitro, in reactions with Atg7, Atg12–Atg5-Atg16, and liposomes generated from *E. coli* polar lipids as a source PE, and detected by migration of Atg8 in Coommassie-stained SDS-PAGE gel. **e** Effects of indicated mutants in Atg3 catalytic domain-E123IR interface on E3-independent Atg8 lipidation in vivo, as detected by western blot for Atg8 after 4 h starvation of the YCY131 multi-Atg knockout strain of *S. cerevisiae* expressing Atg7, Atg10, Atg8∆R (activated in absence of Atg4) and either WT or mutant HA-tagged Atg3

Interestingly, the activated orientation of the catalytic center was observed previously in structures of full-length Atg3 when bound to Atg7 (Supplementary Fig. 4b)^40,43^. Although interpreted as stemming from indirect Atg7 interactions with the backside of Atg3′s catalytic domain^40,43^, and/or from the high pH of the crystallization conditions^35^, reinterpretation of the crystal structures with the knowledge that the Atg3^E123IR^ element binds Atg3′s catalytic domain in solution leads to a prediction: interactions with Atg3^E123IR^ would allosterically restrict Atg3′s catalytic domain. However, displacement of the Atg3^E123IR^ element, either by deleting the FR domain (Fig. 5d), or upon binding to the E1 Atg7 during formation of the Atg3~Atg8 intermediate (Supplementary Fig. 4b), or upon binding to the Atg12–Atg5 portion of E3 for the lipidation reaction^35^, would enable the active conformation. Nonetheless, relevance for the lipidation would depend on the Atg3^E123IR^ element binding in the context of an Atg3~Atg8 complex. We tested this concept by NMR. First, a stable proxy for Atg3^∆FR^–Atg8 was made with a disulfide bond between a Cys replacement for the C-terminal Atg8 residue and the catalytic Cys in Atg3^∆FR^ (Atg3^∆FR^–S–S–Atg8). Upon adding this to the ^15^N-labeled Atg3^FR^, we observed the similar chemical shift perturbations in [^15^N, ^1^H] TROSY spectra of ^15^N-labeled Atg3^FR^ (Fig. 6a) as for Atg3 alone (Fig. 4d).

If binding to the Atg3^E123IR^ would be inhibitory, then mutations in catalytic domain residues that mediate this interaction and that are not needed for other Atg3 functions should stimulate the lipidation reaction in the absence of E3. Therefore, based on the crystallographic (Fig. 5e) and NMR data (Fig. 4f), we generated several Ala substitutions designed to disrupt the autoinhibitory interactions as follows: Asp133, which is the only residue in the Atg3^E123IR^ that does not bind Atg7 and thus could form an Atg3~Atg8 intermediate, interacts with the HR in full-length Atg3; Arg251 and Arg254 in the HR; Lys292, Thr295, and Ser296 in the Atg3^cat^ domain; and Gln302, His303, and Asp304 or Glu308 and Trp310 at the extreme C-terminus (Fig. 6b). These mutations mimicked effects of adding E3 to the in vitro reactions lacking E3: relative to wild-type Atg3, they activate the Atg3~Atg8 thioester-bonded intermediate (Fig. 6c and Supplementary Fig. 5) and the lipidation reaction (Fig. 6d), but are either not affected or defective for E3-dependent activity (Fig. 2b and Supplementary Fig. 6c). The defect for one mutant in E3-dependent activity can be explained due to its location in the E123IR (D133A), but other mutants do not map to a region presently known to be required for activity, although they may reflect an activated conformation of the Atg3~Atg8 intermediate. If this were the case, then the activation observed in the absence of E3 may even be lower than theoretically possible.

Notably, increased Atg3~Atg8 discharge was not observed upon adding liposomes together with the isolated Atg7^NTD^ that like E3 also binds the Atg3^E123IR^ (Supplementary Fig. 5). Thus, releasing the Atg3^E123IR^ from the Atg3^cat^ domain may not be sufficient for activating the lipidation activity of the Atg3~Atg8 intermediate. A potential difference between binding of the Atg7^NTD^ vs. Atg12–Atg5, or the effects of E3-mimicking mutations, would be if the latter alter internal structural dynamics of the Atg3~Atg8 intermediate so as to increase susceptibility to nucleophilic attack.

To test the effects of the activating mutants in vivo, it is necessary to examine Atg8 lipidation in the absence of the proteins comprising the E3. We also wished to minimize confounding effects of Atg8, Atg3, and the Atg12–Atg5-Atg16 complexes interacting with many proteins associated with autophagy and nonautophagy-related pathways. Thus, we generated a minimal system for examining Atg8 lipidation in vivo, with a plasmid expressing Atg7, HA-tagged versions of Atg3, and the processed form of Atg8 in a yeast strain in which these and 21 other autophagy (Atg) genes have been deleted (MKO)^55^. Atg8 lipidation was markedly increased with the majority of the mutants (Fig. 6e), which is remarkable given the complexity of events required for the reaction and the large number of other proteins that interact with Atg3 and Atg8 in vivo.

### Extensive surfaces are required for Atg3~Atg8 activation

**Fig. 7:**
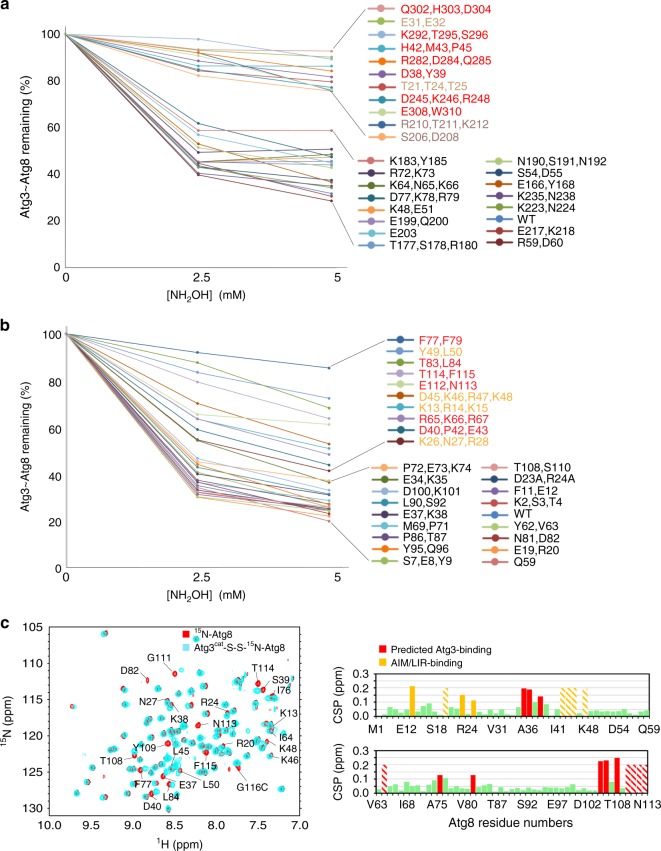
Extensive surfaces of Atg8 and the Atg3 catalytic domain are required for activation of the Atg3~Atg8 intermediate. **a** E3-dependent activation of Atg3~Atg8 intermediate testing roles of indicated surfaces through multiple-Ala scanning mutagenesis over Atg3′s catalytic domain. Quantification is of WT or indicated mutant versions of Atg3~Atg8 remaining after 2.5 min as a function of NH_2_OH concentration in pulse-chase assays, with Atg12–Atg5-Atg16 as E3. **b** E3-dependent activation of Atg3~Atg8 intermediate testing roles of indicated surfaces through multiple-Ala scanning mutagenesis over Atg8, performed as in (**a**). **c** Effects of covalent Atg3 complex formation with Atg8, as detected by comparing ^15^N, ^1^H] TROSY spectra of ^15^N-labeled Atg8 G116C (red) alone and disulfide-bonded complex with Atg3^cat^ as a proxy for Atg3^cat^–Atg8 intermediate (cyan), with chemical shift perturbations per Atg8 residue shown below. Stripes indicate resonances with line-broadening due to intermediate exchange

**Fig. 8:**
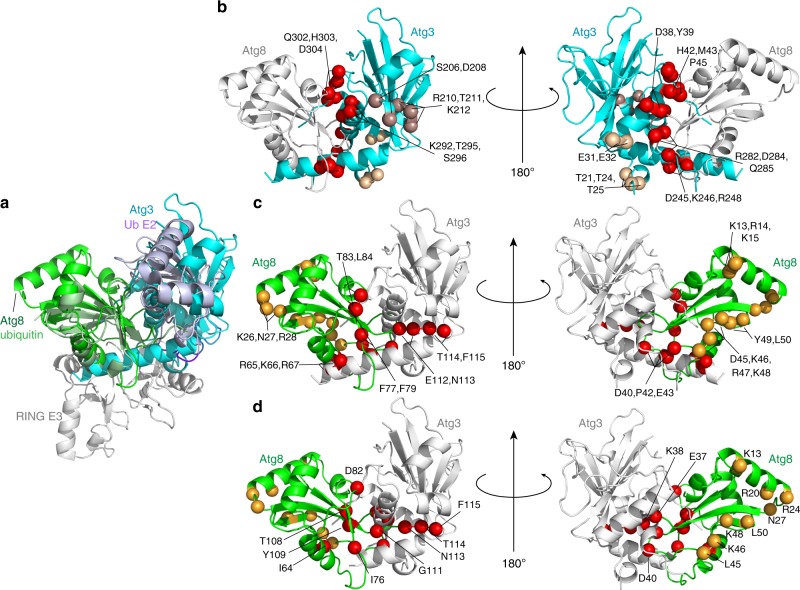
Modeling of the active conformation of Atg3~Atg8 intermediate. **a** Generation of model for a potential closed E2~Ubl conformation for Atg3~Atg8, with structures of Atg3^∆NFR^ (PDB 6OJJ, from this study) and Atg8 (PDB 2ZPN) superimposed on E2 and Ub, respectively, in a RING E3–E2–Ub complex (PDB 4AP4). **b** Sites of Atg3 mutations impairing E3-dependent activation of Atg3~Atg8, mapped on model for closed conformation. Red—residues corresponding to E2–Ub interface in closed conformation; wheat—residues corresponding to RING E3-binding site; bronze—residues in catalytic segment. **c** Sites of Atg8 mutations impairing E3-dependent activation of Atg3~Atg8, mapped on model for closed conformation. Red—residues corresponding to E2–Ub interface in closed conformation; orange—residues corresponding to AIM/LIR-binding site. **d** Atg8 residues showing chemical shift perturbation one standard deviation above the mean upon covalent complex formation with Atg3, mapped on model for closed conformation. Red—residues corresponding to E2–Ub interface in closed conformation; orange—residues corresponding to AIM/LIR-binding site

To identify the portions of Atg8 and the Atg3 catalytic domain contributing to the activated state, we performed mutagenesis. E3-dependent discharge was monitored for 28 mutant versions of Atg8 and 27 mutant versions of Atg3, each having one to four Ala replacements for surface residues (Fig. 7a, b and Supplementary Fig. 6a, b). The Atg8 mutations together probed the majority of its surface, while Atg3 mutations encompass the catalytic domain that is structurally homologous to canonical E2s and the short C-terminal extension. A strikingly large fraction of the mutants affected activation of Atg8 discharge. Notably, the majority of the most defective mutants were also defective for Atg8 lipidation in vitro (Supplementary Fig. 7a, b) and in vivo (Supplementary Fig. 7c). For Atg3, the defective mutants map to a large continuous surface encompassing Atg3′s α1-, HR-, and α5- (large central), α4- (adjacent to the catalytic center) helices, and C-terminal extension (Fig. 8b). For Atg8, the mutations map to two major surfaces. One corresponds to the canonical UBL hydrophobic patch and C-terminus, and the other to the Atg8-specific groove that binds AIM/LIR sequences in partner proteins (Fig. 8c).

We hypothesized that some of the mutational defects could reflect impaired interactions between Atg8 and Atg3 in the covalent intermediate. Thus, we performed NMR considering that prior studies of intrinsically active E2–UB intermediates showed chemical shift perturbations indicating noncovalent interactions between the covalently linked E2 and UB moieties. Thus, we compared [^15^N, ^1^H] TROSY spectra for ^15^N-labled Atg8 harboring a C-terminal Cys alone vs. in a disulfide-bonded complex linked to the active site of unlabeled Atg3^cat^—i.e., in a stable proxy for the Atg3^cat^–Atg8 intermediate (Fig. 7c).

The striking similarity between the locations of Atg8 NMR resonances shifting upon covalent complex formation with Atg3 (Fig. 8c), and of mutants impairing E3-activated discharge from Atg3 (Fig. 8b) raise the possibility of noncovalent interaction in a so-called closed conformation. We used structural modeling to consider potential parallels between the activated Atg3~Atg8 intermediate and a RING E3–E2–UB complex as representative. First, the structure of Atg3 was superimposed with the homologous region of the E2 UBE2D1 in the intermediate, and then the UB-fold domain of Atg8 was docked onto the linked UB (Fig. 8a). Next, the locations of mutations impairing activity, and NMR chemical shift perturbations in the Atg3^cat^–Atg8 complex, were analyzed, and found to be in striking concordance with the corresponding interface residues in canonical E2–UB intermediates (Fig. 8b, c, d). The notable exceptions were the AIM/LIR docking site in Atg8, Atg3 residues corresponding to E3-binding regions of canonical E2s, and the extreme C-terminus of Atg3. These regions are not shared between Atg3 and canonical E2s, and the latter region is not fully visible in the crystal structures presumably due to potential to adopt different conformations. Nonetheless, the differences for the autophagy-specific regions that are observed between the crystal structures (Supplementary Fig. 4c), and in NMR chemical shift perturbations (Fig. 4c), for the E123IR-bound and other forms of the Atg3 catalytic domain are consistent with roles in allosteric regulation. Indeed, in using the Atg3^∆FR^ crystal structure for the structural model, the C-terminal residues of Atg3 approach the docked Atg8. On this basis, it is tempting to speculate that this sequence, Met-Glu-Gly-Trp, would adopt a reverse AIM/LIR motif anchoring Atg8–Atg3 in a closed conformation. Concordantly, mutation of these residues has amongst the most deleterious effects (Fig. 7a and Supplementary Figs. 6a, 7a), although future studies will be required to visualize the structure of the E3-activated Atg3~Atg8 intermediate.

## Discussion

Here, we demonstrated that Atg3 is restrained by an E123IR element within its 70-residue FR bracing the active site; this brace establishes regulation by interacting with E1 (Fig. 4a)^42^, E2 (Fig. 4c, d, f), and E3 (Fig. 3a, c); and that a key function of the autophagy E3 Atg12–Atg5-Atg16 is to bind this brace to activate the intrinsic reactivity of the thioester-bonded Atg3~Atg8 intermediate (Fig. 4b). This unprecedented allosteric mechanism underlying E2 activation—revealed by our NMR, crystallographic, and mutational data, taken together with prior studies—addresses the longstanding question of how Atg8 lipidation is activated by an E3 that lacks sequence and mechanistic conservation with canonical conjugation enzymes. Notably, E1-binding to the E123IR element is not sufficient to activate the Atg3~Atg8 intermediate (Supplementary Fig. 5), suggesting additional roles for E3. Meanwhile, NMR data raise the possibility that Atg3′s active site residues undergo conformational motions on a different time-scale from the rest of catalytic domain, which could impact enzymatic activity (Fig. 4c). Taken together, we speculate that E3 binding to the Atg3~Atg8 intermediate broadly influences dynamic or structural features of Atg3′s catalytic domain that in turn bias the active site in favor of the reactive conformation. Indeed, the need for E3-binding to activate the Atg3~Atg8 intermediate in vitro is partially ameliorated by mutations that map to the surfaces in and adjacent to the Atg3^cat^–Atg3^E123IR^ interface (Fig. 6c, d and Supplementary Fig. 5). Physiological relevance is supported by such mutants also activating E3-independent lipidation in vivo (Fig. 6e).

**Fig. 9:**
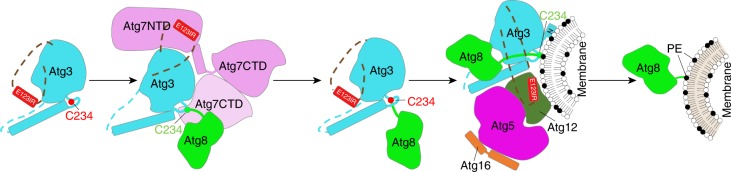
Schematic model for allosteric regulation of Atg3 activity through E123IR interactions across the lipidation cascade. The autophagy E2 Atg3 is autoinhibited by Atg^E123IR^. When Atg3 encounters E1 Atg7, E123IR is relocated upon binding to Atg7^NTD^, thereby triggering rearrangement of the Atg3 catalytic core to activate Cys234 for attacking the Atg7–Atg8 intermediate. Atg8 is transferred from Atg7 to Atg8, producing the thioester-bonded Atg3~Atg8 intermediate. Relieved from Atg7, Atg3^E123IR^ protects the Atg3~Atg8 intermediate from wayward discharge to errant nucleophiles. The E3 (Atg12–Atg5-Atg16) binds E123IR and further activates the Atg3~Atg8 intermediate for nucleophilic attack. Numerous interactions with membranes place this complex in proximity to PE for the lipidation reaction

Although future studies will be required to visualize the downstream steps in the reaction cascade, we propose that Atg8 lipidation involves Atg7, Atg3, and Atg12–Atg5-Atg16 toggling their common interacting region in Atg3 as follows: (1) Prior to encountering the Atg7–Atg8 intermediate, the Atg3^E123IR^ binds to the Atg3 catalytic domain to protect the active site Cys from inactivation through with cellular small molecules. (2) Atg7′s N-terminal domain binds to Atg3′s E123IR element. This both mediates E1–E2 interactions and conformationally activates Atg3′s Cys to receive Atg8 from Atg7. (3) Following formation of the Atg3~Atg8 intermediate, Atg3′s E123IR element could re-engage the Atg3 catalytic domain to prevent Atg8 mis-ligation to a nonspecific nucleophile. We speculate that this may also prevent the extreme C-terminal residues of Atg3 from prematurely adopting a conformation that contributes to the activate conformation of the Atg3~Atg8 intermediate. (4) Binding with relatively higher affinity, the Atg12–Atg5 portion of the autophagy E3 subsequently dislodges Atg3′s E123IR, which enables structural remodeling of the active site loop from a sequestered position to an exposed conformation where the catalytic Cys is juxtaposed with side chains that are crucial for Atg8 lipidation (Fig. 9). Apparently, Atg12–Atg5 further activates the lipidation reaction in a manner involving extensive surfaces from both Atg3 and Atg8 in the Atg3~Atg8 intermediate. Due to parallels to RING and SUMO E3 activation of canonical E2–UBL intermediates^45–47^—both in stimulating intrinsic reactivity and in the locations of the E2 and UBL residues involved—we speculate that the Atg12–Atg5 autophagy E3 mediates activation in part by promoting interactions between Atg3 and Atg8 in the Atg3~Atg8 intermediate, although details of the interaction remain to be discovered and may be distinct for the autophagy enzymes.

Why might the autophagy UBL pathway involve such elaborate allosteric control? It seems likely that key features of the ultimate target, and intrinsic capabilities of the Atg3~Atg8 intermediate, impose distinctive requirements for regulation. First, the substrate PE is among the most abundant core glycerophospholipids. Found in several organelles, PE’s widespread accessibility differs from canonical UB and UBL substrates, which typically only are licensed for modification after a regulatory event such as a post-translational modification directs E3 binding. Second, through its N-terminal membrane-curvature sensing helix, Atg3 itself can bind the substrate PE^37^. The active site of Atg3~Atg8 may require protection to prevent inopportune discharge to PE, and this is best ensured by coming from the intermediate itself. Indeed, when conformationally activated by E3-mimicking mutations, Atg3~Atg8 cannot only catalyze the lipidation reaction and in vivo, but also robustly discharges Atg8 to nonspecific nucleophiles even in the absence of E3. Thus, our data provide a mechanism ensuring that Atg8 discharge is coupled to binding to E3, which in turn establishes targeting specificity through several features, including binding to cargo receptors^56^, and to membranes^27,31^. The Atg16 moiety in the autophagy E3 could subsequently engage the lipidated Atg8 to serve as a scaffold during autophagosome biogenesis^32^.

Allosteric E2 activation is emerging as a common property of E3 ligases^45–47^. It is now well-recognized that many E3 catalytic domains stabilize a closed conformation that renders E2–UB and E2–UBL intermediates susceptible to nucleophilic attack, while other E3 domains mediate substrate targeting. Many E2s are further activated allosterically, through their backside beta-sheets binding to noncatalytic E3 domains. These effects have largely been characterized for indirectly increasing binding to E3 RING domains. However, some E3 or E3-associated domains also trigger conformational changes at E2 active sites. For example, the membrane E3-associated Cue1p binds to Ubc7p’s backside, and indirectly modulates the active site, tens of angstroms away^57^. Irrespective of whether E2 cysteines require protection from errant nucleophilic attack, or coordination of catalytic activation with other E3 functions, it seems likely that allosteric modulation of E2 active sites serves as a failsafe ensuring UB and UBL discharge at appropriate targets. The autophagy E2 Atg3 now surfaces as an ultimate case, with its brace self-imposed until directly lifted by the E3 to drive autophagy.

## Methods

### Protein sequence alignment

ScAtg3 and HsATG3 protein sequence alignment are done by CLUSTALW online.

### Protein expression and purification

Detailed methods for cloning, expressing and purifying yeast proteins Atg8, Atg3 (and their mutants or truncated versions), and Atg16 (residues 1–46) were described previously^42,43,58^. For Atg7 proteins, full-length Atg7 or a fragment containing residues 1–289 (NTD) was inserted into the pGEX-4T-1 vector following the Gibson Assembly protocol^59^, and transformed into BL21 competent *Escherichia coli*; protein expression and purification protocols for Atg7 WT and mutants resemble those for Atg3 and Atg8. To obtain purified E3 complex, N-term 6X His-tagged Atg12 100–186 and untagged Atg5 1–284 were inserted into the two multiple cloning sites (MSCs) of pRSF-Duet-1, respectively, whereas Atg7 1–630 and Atg10 were inserted into the MSCs of pET-Duet-1. The two plasmids are co-transformed into BL21 competent *E. coli* to produce His-Atg12–Atg5. Bacteria culture were grown at 37 °C to reach O.D. 1.5, followed by 0.6 mM IPTG induction at 23 °C overnight. Cells are harvested in 1× phosphate-buffered saline, 400 mM NaCl and lysed by sonication. Ni-NTA resin (Qiagen) was incubated with lysate for 1 h then washed 3 times with the same buffer. His-Atg12–Atg5 were eluted by buffer with 200 mM imidazole and immediately purified by cation exchange chromatography on an S column (GE Healthcare). Purified his-Atg12–Atg5 were combined with Atg16 1–46 in a 1:2 molar ratio and further purified by size-exclusion chromatography on a Superdex-200 column (GE Healthcare) to harvest peak factions containing the final product of His-Atg12–Atg5-Atg16.

### Crystallization and data collection

Totally, 1 µL purified Atg3^∆NFR^ protein sample was manually loaded onto a glass hanging-drop cover and overlaid with 1 µL of precipitant solutions. Crystal was harvested from 100 mM HEPES pH 7.2, 150 mM l-malic acid, 9% PEG3350. Crystals were allowed to grow at 4 °C for 5–7 days to reach their maximum sizes. All crystals were harvested by loops with 25% glycerol in addition to growth solution, and stored in liquid nitrogen for 2 days until data collection.

### Data processing and structure refinement

Data for Atg3^∆NFR^ was collected at Advanced Photon Source (APS) Ser-CAT beamline 22-BM. The Dataset was created from single crystals, and was processed and scaled in HKL2000. Phase was solved by molecular replacement. Search model for Atg3^∆NFR^ was modified from Atg3^FL^ (PDB 2DYT) with Atg3^E123IR^ manually removed. Model was built in Coot^60^, and refinements were primarily performed in Refmac^61^ and finalized in Phenix^62^.

### Simulated annealing omit map generation

In order to avoid the model bias, we generated a simulated annealing omit map for the catalytic cysteine region. First, residue 231–237 in the final-refined model was deleted; then, the model with deletion was subjected to 3 rounds of refinement in Phenix^62^ with simulated annealing running from 5000 to 300 K in 50 steps. Finally, the Fo–Fc map was shown at 3*σ* level along with the deleted residues in the model.

### Atg3 C234 and Atg8 G116C disulfide cross-linking

All the cysteines except C234 within Atg3 were mutated to alanines. 800 µM Atg8 K26P C22V G116C was reduced by 20 mM DTT, desalted into buffer 1 (20 mM HEPES pH 7.5, 50 mM NaCl), and immediately mixed with 1:1 v/v buffer 2 (20 mM HEPES pH 7.5, 50 mM NaCl, 2.5 mM 2,2′-dipyridyldisulfide (DPS). DPS stock was pre-dissolved in a minimal amount of DMSO), incubated at room temperature (RT) for 20 min, and desalted again into buffer 1. Meanwhile, 500 µM Atg3 was reduced by 20 mM DTT and desalted into buffer 1, immediately mixed with DPS-Atg8 at a 1:2 molar ratio, and incubated at RT for 1 h. Crosslinking product was purified by anion exchange chromatography on Q column (GE Healthcare) and size-exclusion chromatography on a Superdex-200 column (GE Healthcare).

### NMR spectroscopy

All the experiments were conducted at 25 °C with protein samples dissolved in 20 mM MES pH 6.5, 100 mM NaCl, and 10% v/v D_2_O on either a Bruker Avance 600-MHz or 700-MHz or 800-MHz spectrometer equipped with a 5-mm triple-resonance cryoprobe and a single-axis pulse field gradient. For resonance assignments of backbone atoms, 3D triple-resonance experiments were measure either with 0.4 mM ^13^C, ^15^N, ^2^D-labeled or ^13^C, ^15^N-labeled Atg3^cat^, 0.75 mM ^13^C, ^15^N-labeled Atg3^FR^ 86–159, and 0.4 mM ^13^C, ^15^N-labeled Atg8 K26P, C33V, G116C. The backbone assignment of Atg3^cat^ was carried out using 3D HNCACB, HNcoCACB, HNCO, HNcaCO along with ^15^N-resolved [^1^H, ^1^H] NOESY spectra. The backbone of Atg3^FR^ 86–159 fragment peptide was assigned using 3D HNCACB, CACBcoNH, HNCO, HNcaCO along with HNcocaNH spectra. The backbone assignment of Atg8 K26P, C33V, G116C was carried out using 3D HNCACB, HNcoCACB, HNCO, HNCA and HNcoCA along with ^15^N-resolved [^1^H, ^1^H] NOESY spectra. Spectra were processed with Topspin 3.5 or nmrPipe and analyzed using CARA. All chemical shift perturbation analyses were done using [^15^N, ^1^H] TROSY spectra, and the perturbations were calculated using the formula CSP (ppm) = ((δH)^2^ + 0.2*(δN)^2^)^0.5^. Titration experiments were done using 0.1 mM ^15^N-labeled Atg3^FR^ 86–159 (or its I132D, L135D, I136D mutant) titrated with 0–150 µM Atg12–Atg5 (Fig. 3a), 0–500 (Fig. 4d) or 0–625 μM Atg3^∆FR^ (Supplementary Fig. 3a), or 0 and 0.2 mM unlabeled Atg3^∆FR^ C234–Atg8 K26P, C33V, G116C disulfide complex (Fig. 6a); 0.1 mM ^15^N-labeled Atg3^cat^ was titrated with 0 or 4.4 mM unlabeled Atg3^FR^ 123–147 (Fig. 4c). Also spectrum from 0.1 mM ^15^N-labeled Atg8 was compared with 0.1 mM disulfide complex of unlabeled Atg3^cat^ cross-linked via C234 to ^15^N-labeled Atg8 K26P, C33V, G116C (Fig. 7c). The CSP value is used in the titration plots to get the *K*_d_ values using the equation: *y* = (*y*0 + *x*)/(*x* + *K*_d_), where *x* is the concentration and *K*_d_ is the dissociation constant and *y*0 is a constant.

Since most of the resonances in Atg3^FR^ mutant [I132D, L135D, I136R] have the same chemical shifts as that of Atg3^FR^, the assignment of Atg3^FR^ was used to assign the resonances of Atg3^FR^ mutant (Supplementary Fig. 3a). The assignment of the mutated residues and the neighboring ones that shifted the most ambiguously shifted to positions where new peaks appeared.

### Liposome preparation

2.5 mg of *E. coli* polar lipid extract (Avanti) was dissolved in chloroform, solvent evaporated using a nitrogen stream, and re-dissolved in 250 µL 20 mM HEPES pH 7.0, 150 mM NaCl buffer. Product was passed through 100 nm nuclepore membrane (Whatman) installed in a mini extruder (Avanti) 15 times to obtain 10 mg/mL homogenized liposomes in solution.

### In vitro pulse-chase assay for Atg3~Atg8 discharge to NH_2_OH

Shortly before the starting the experiment, Atg3 and mutants were reduced with 20 mM DTT and desalted by Zeba columns (Thermo Fisher Scientific) into reaction buffer. In the pulse step, 5 µM Atg7, 20 µM Atg3, 40 µM Atg8, 1 µM ATP, and 1 µM MgCl_2_ were mixed in 25 µL 20 mM HEPES pH 7.0, 150 mM NaCl buffer. After 1 h, pulse reactions were quenched by adding Apyrase. In chase step, 5 µL of each pulse product was incubated with 5 µL chase solution containing 5 µM Atg12–Atg5-Atg16 and a course of NH_2_OH at RT for 2.5 min, or with liposome at a final concentration of 2 mg/mL added in a timely manner. Samples were analyzed by 4–12% Bis–Tris Protein Gels (Thermo Fisher Scientific) and stained with Coomassie Brilliant Blue. All raw data are available in the source data file.

### In vitro assay for Atg8 lipidation

Atg3 and mutants were reduced and desalted as described in the pulse-chase assay. Reactions were performed at 30 °C in 25 µL volumes of 20 mM HEPES pH 7.0, 150 mM buffer, with 1 µM Atg7, 2 µM Atg3, 5 µM Atg8, 5 µM Atg12–Atg5-Atg16, 1 mM ATP, 1 mM MgCl_2_ and 1 mg/mL liposome, and were monitored in timely manner. Totally, 6 µL of each reaction was mixed with 6 µL 2× protein loading buffer. Samples were analyzed using a by 15% urea gel at 4 °C and stained with Coomassie Brilliant Blue. All raw data are available in the source data file.

### Yeast strains and vectors

Yeast strains used in this study are listed in the table below. The XLY161 and the YCY131^55^ cells were transformed with pRS416 vectors harboring HA-tagged Atg3 (WT or mutants); the YCY131 cells were co-transformed with pRS414–Atg7–Atg10–Atg8∆R. For nutrient-rich conditions, yeast cells were grown in synthetic minimal (SMD; 0.67% yeast nitrogen base, 2% glucose, and auxotrophic amino acids and vitamins as needed) medium. For nitrogen starvation, cells were first cultured in appropriate SMD medium to mid-log phase; they were then shifted to the SD-N medium (0.17% yeast nitrogen base without ammonium sulfate or amino acids, 2% glucose). The XLY161 cells were cultured in SMD-Ura to mid-log phase before they were shifted to SD-N medium for 2 h. The YCY131 cells were cultured in SMD-Ura-Trp to mid-log phase before they were shifted to SD-N medium for 4 h. All raw data are available in the source data file.

### Western blot

One OD of each of the yeast cultures was harvested and lysed in 50 μl MURB (50 mM NaH2PO4, pH 7.0, 2.5 mM MES, pH 7.0, 1% SDS, 3 M urea, 0.5% β-mercaptoethanol, 1 mM NaN3, 0.2 μg/μl bromophenol blue) by vortexing them with glass beads at 4 °C for 5 min. The samples were then heated at 70 °C for 10 min before they were loaded onto and run in SDS-PAGE gels, followed by transferring onto polyvinylidene fluoride membranes. The membranes were blocked with 5% skim milk, probed with primary antibodies (1:3000, Atg8; 1:10,000, Dpm1; 1:3000, HA; and 1:10,000, Pgk1), and then probed with horseradish peroxidase (HRP)-coupled secondary antibodies (1:10,000, anti-rabbit and anti-mouse antibodies). Atg8 antiserum was generated by injecting purified yeast Atg8 peptides into rabbits; Dpm1 antibody is purchased from Molecular Probes (catalog# A-6429); antibody to the HA epitope is from Sigma (catalog# H3663); Pgk1 antibody is a generous gift from Dr. Jeremy Thorner, University of California, Berkeley. Goat anti-rabbit HRP sencondary antibody is from Fisher (catalog# ICN55676), and rabbit anti-mouse HRP sencondary antibody is from Jackson ImmunoResearch (catalog# 315-035-003). Chemiluminescence images were obtained by developing films in a dark room. The standard-sized protein bands from the protein ladder lanes on the blots were correspondingly hand-drawn using a pen onto the developed films. The hand-drawn protein ladders were not shown in the figures, but they can be found in the source data file.

### Reporting summary

Further information on research design is available in the Nature Research Reporting Summary linked to this article.

## Supplementary information

Supplementary Information

Peer Review

Reporting Summary

Source Data

## Data Availability

The coordinates and structure factors for Atg3^∆NFR^ have been deposited to the RCSB Protein Data Bank with ID 6OJJ. The resonance assignments for Atg3^cat^, Atg3^FR^ (residues 86–159), and Atg8 have been deposited to the Biological Magnetic Resonance Data Bank with the IDs 27922, 27923, and 27924, respectively for immediate release upon paper publication. The source data for Figs. 1b, 2b, 2c, 4b, 6d, 6e, and Supplementary Figs. 1b, 1c, 2, 5, 6a, 6b, and 7a–c are provided as a Source Data file. Other data are available from the authors upon request.
