# Supplementary material for: A switch element in the autophagy E2 Atg3 mediates allosteric regulation across the lipidation cascade
Source: Nat Commun. 2019 Aug 9;10:3600. doi: 10.1038/s41467-019-11435-y (PMC6689050; doi:10.1038/s41467-019-11435-y)
Supplement: Supplementary file 1 — Supplementary Information [file 41467_2019_11435_MOESM1_ESM.pdf]

Supplementary Information for: A switch element in the autophagy E2 Atg3 mediates allosteric regulation across the lipidation cascade

Yumei Zheng, Yu Qiu, Christy R. R. Grace, Xu Liu, Daniel J. Klionsky, Brenda A. Schulman

Contents

Supplementary Figure 1: Lack of sequence conservation for Atg3 FR yet functional conservation of FR-binding site on Atg12 within Atg12–Atg5 E3 module.

Supplementary Figure 2: Alanine scan within FR of Atg3 for E3-dependent activation of Atg3~Atg8 intermediate.

Supplementary Figure 3: Mutations in E123IR hydrophobic residues significantly impair E123IR-Atg3 interaction.

Supplementary Figure 4: Structural remodeling of the Atg3 catalytic core upon displacement or removal of the E123IR element.

Supplementary Figure 5: Mutations in interface between Atg3's E123IR element and catalytic domain activate the Atg3~Atg8 intermediate.

Supplementary Figure 6: Alanine scan within Atg3^{cat} and Atg8 by NH_2OH discharge assays.

Supplementary Figure 7: Alanine mutants within Atg3cat and Atg8 examined by Atg8-lipidation assays in vitro and in vivo.

Supplementary Table 1: Data collection and refinement statistics.

a

```
                                  N-term
ATG3_YEAST    ------MIRSTLSSWREYLTPITHKSTFLTTGQITPEEFVQAGDYLCHMFPTWKWNEESS
ATG3_HUMAN    MQNVINTVKGKALEVAEYLTPVLKESKFKETGVITPEEFVAAGDHLVHHCPTWQWATG-E
                     ::..  .  *****: ::*.*  ** ******* ***:* *  ***:*    .
                                                           FR
ATG3_YEAST    DISYRDFLPKNKQFLIIRKVPCDKRAEQCVEVEGPDVIMKGFAEDGDEDDVLEYIGS--E
ATG3_HUMAN    ELKVKAYLPTGKQFLVTKNVPCYKRCKQMEYSDELEAIIEEDDGDGGWVDTYHNTGITGI
              ::. : :**..****: ::*** **.:*    :  :.*::    **.  *. .  *
                                  E123IR            FR
ATG3_YEAST    TEHVQSTPAGGTKDSSIDDIDELIQDMEIKEEDENDDTEEFNAKGGLAKDMA--------
ATG3_HUMAN    TEAVKEITLENKDNIRLQDCSALCEEEEDEDEGEAADMEEYEESGLLETDEATLDTRKIV
              ** *:. .  ...:  ::* . * :: * ::*.*  * **:: .* * .* *

ATG3_YEAST    ----------------QERYYDLYIAYSTSYRVPKMYIVGFNSNGSPLSPEQMFEDISAD
ATG3_HUMAN    EACKAKTDAGGEDAILQTRTYDLYITYDKYYQTPRLWLFGYDEQRQPLTVEHMYEDISQD
                              * * *****:*.. *:.*::::.*::.: .**: *:*:**** *
                                                                HR
ATG3_YEAST    YRTKTATIEKLPFYKNSVLSVSIHPCKHANVMKILLDKVRVVRQRRRKELQEEQELDGVG
ATG3_HUMAN    HVKKTVTIENHPHLP-PPPMCSVHPCRHAEVMKKIIETVAEG------------------
              : .**.***: *.   .    *:***:**:*** :::.*
                   HR                      C-term
ATG3_YEAST    DWEDLQDDIDDSLRVDQYLIVFLKFITSVTPSIQHDYTMEGW-
ATG3_HUMAN    ---------GGELGVHMYLLIFLKFVQAVIPTIEYDYTRHFTM
                       ...* *. **::****: :* *:*::*** .
```

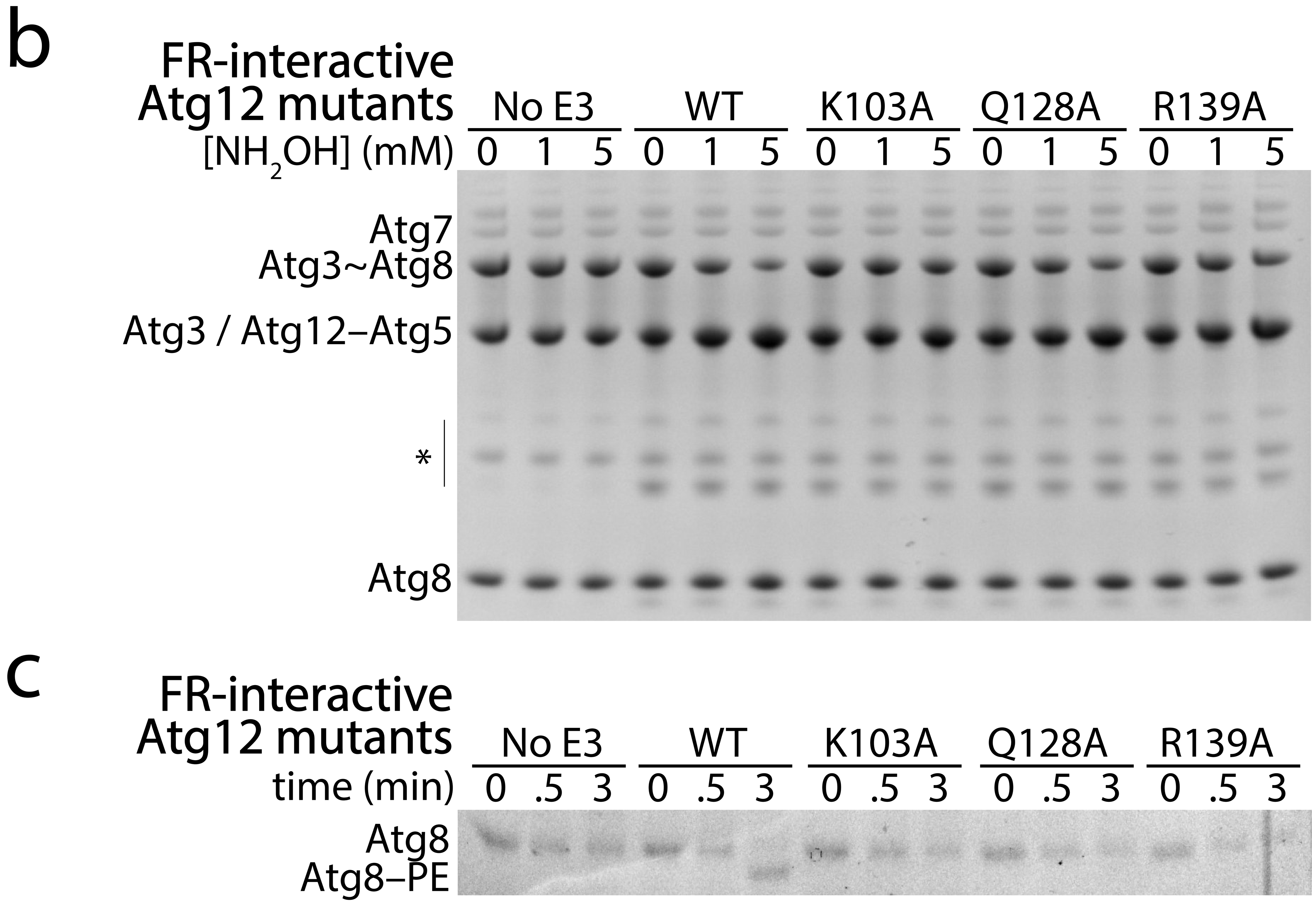

Supplementary Figure 1: Lack of sequence conservation for Atg3 FR yet functional conservation of FR-binding site on Atg12 within Atg12–Atg5 E3 module.

a, Sequence alignment of Atg3 from S. cerevisiae (yeast) and H. sapiens (human). Elements in yeast Atg3 are labeled. **b,** Representative gel image of 3 times repeated assays from Figure 1d. Upon manuscript acceptance, all raw data points will be provided as Source Data. **c,** Effects of indicated Atg12 Ala mutants in predicted FR-binding site from Atg12–Atg5 on Atg8 lipidation in vitro, in reactions with Atg7, Atg12–Atg5-Atg16, and liposomes generated from E. coli polar lipids as a source PE, and detected by migration of Atg8 in Coommassie-stained SDS-PAGE gel.

Atg3 mutants within FR

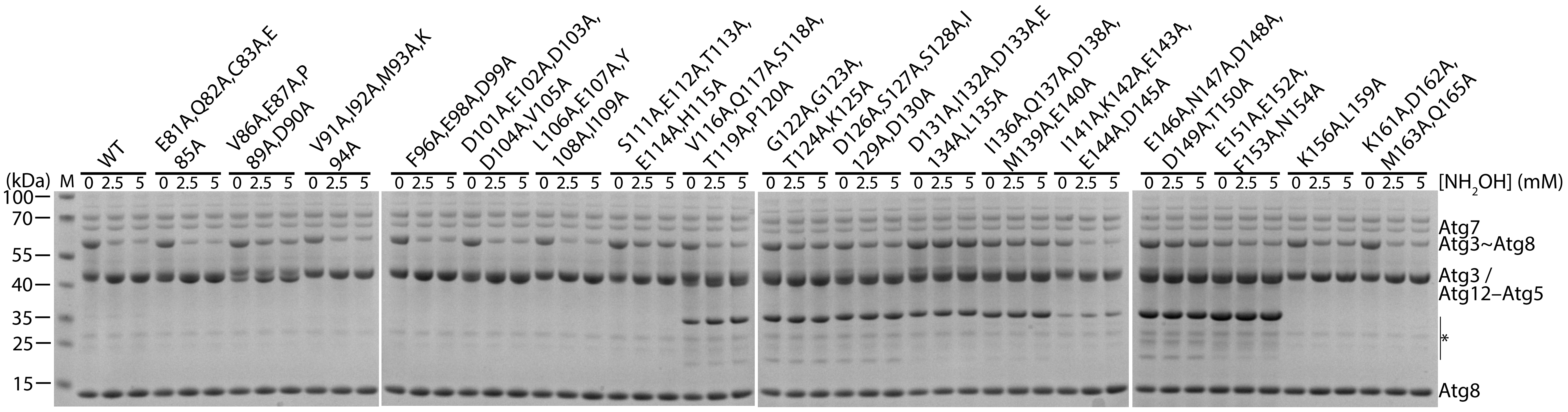

Supplementary Figure 2: Alanine scan within FR of Atg3 for E3-dependent activation of Atg3~Atg8 intermediate.

Representative gel image of assay from Figure 2a.

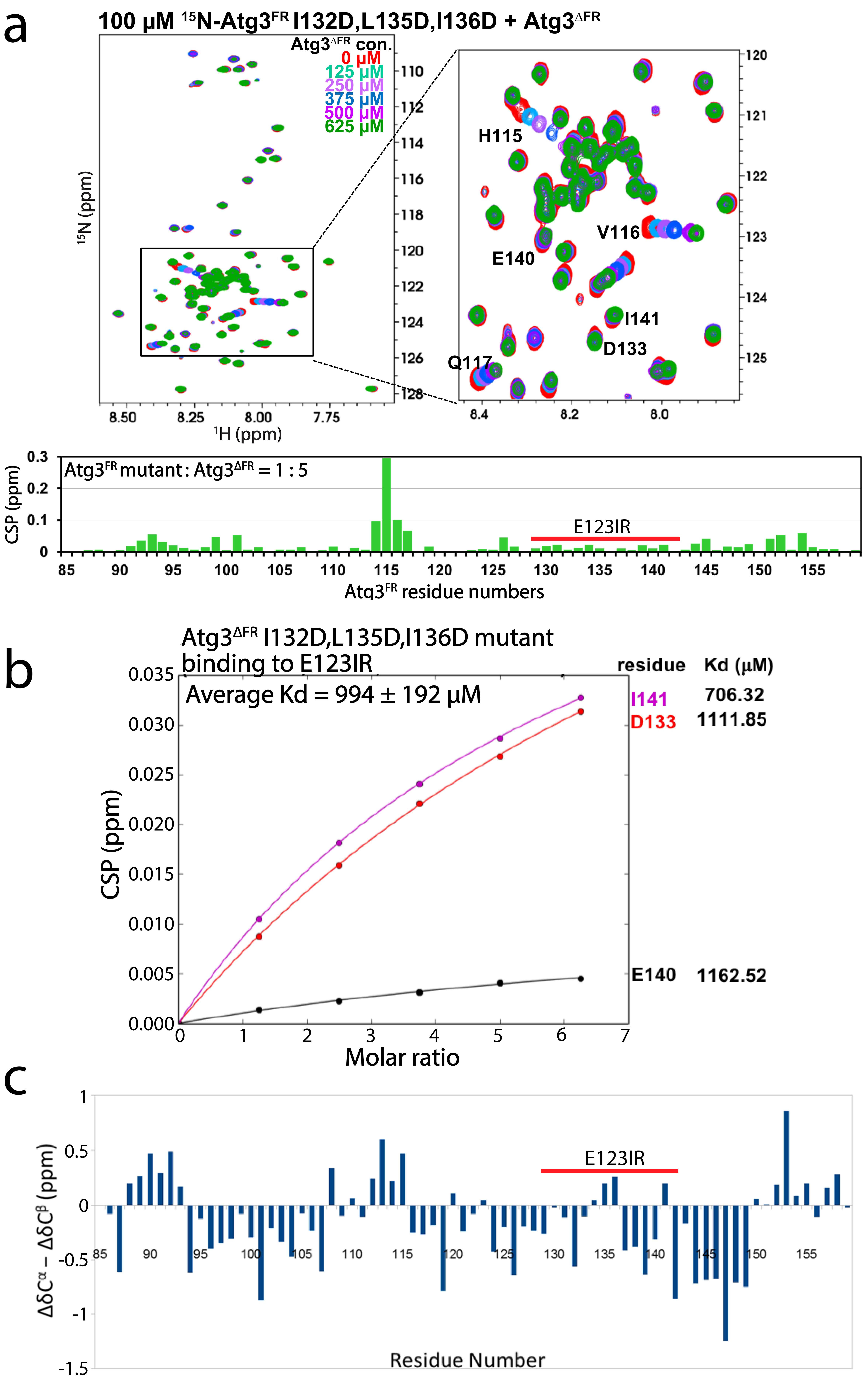

Supplementary Figure 3: Mutations in E123IR hydrophobic residues significantly impair E123IR-Atg3 interaction.

a, [^{15}N, ^{1}H] TROSY spectra of ^{15}N-labeled Atg3^{FR} titrated with increasing concentrations of unlabeled Atg3$^{\Delta FR}$, with chemical shift perturbations (CSPs) plotted per residue shown below for one representative point in the titration (1:5 molar ratio Atg3^{FR} mutant versus Atg3$^{\Delta FR}$). **b,** Calculated binding affinities between Atg3$^{\Delta FR}$ and residues in the I132D,L135D,I136D mutant version of Atg3^{FR}, based on CSPs along the titration. **c,** The ΔδCα – ΔδCβ secondary chemical shifts of the Atg3^{FR} peptide in its free form.

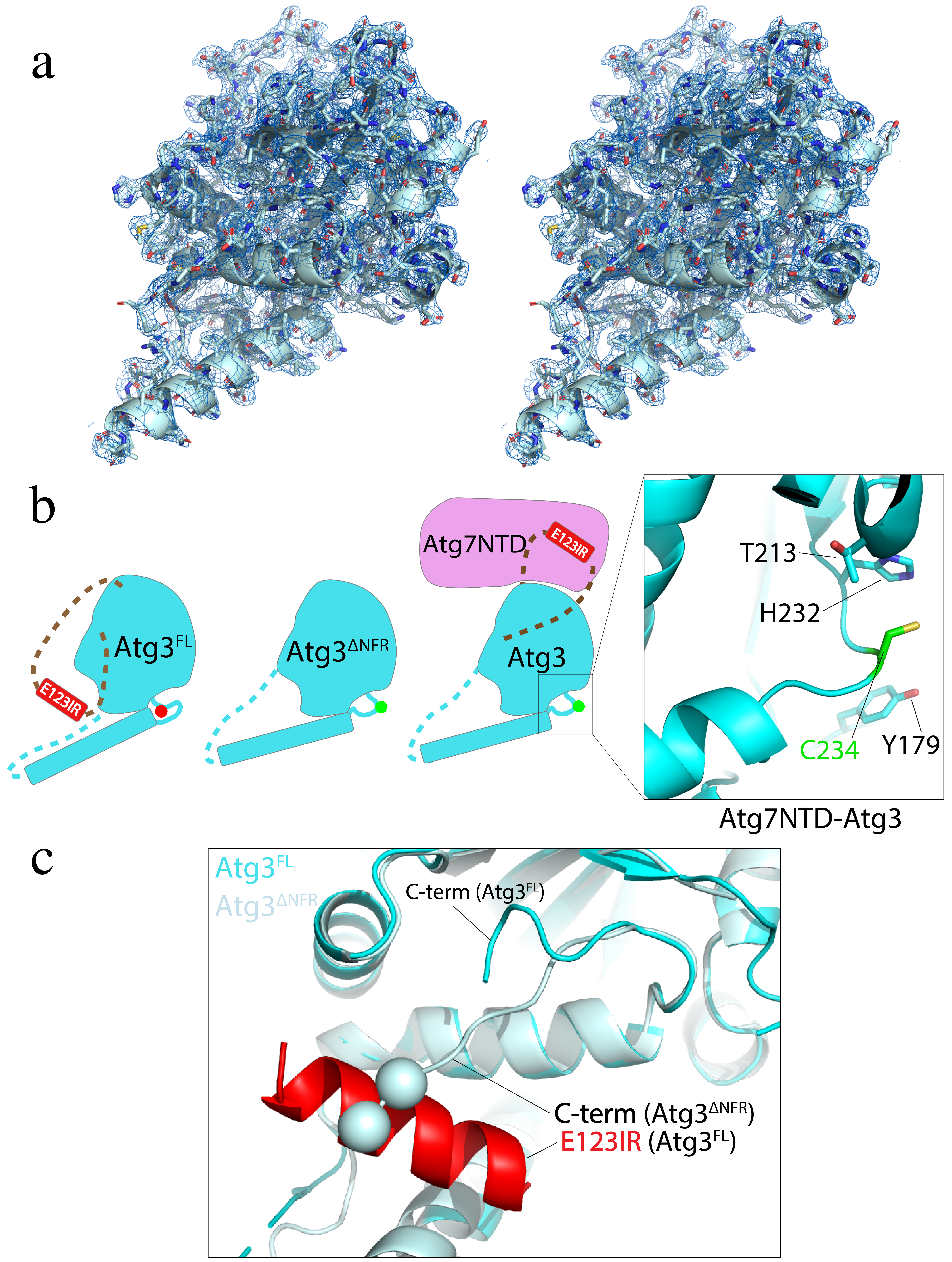

Supplementary Figure 4: Structural remodeling of the Atg3 catalytic core upon displacement or removal of the E123IR element.

a, Stereo view of Atg3$^{\Delta NFR}$ crystal structure in 2F0-Fc density map, σ=1.0. **b**, Schematics of structures of Atg3, with E123IR bound (from Atg3^{FL} PDB 2DYT), with E123IR removed in Atg3$^{\Delta NFR}$ (this study, PDB 6OJJ), and with E123IR dislodged upon binding to Atg7 (PDB 4GSL), with the conformation in the Atg7-bound complex shown to the right for comparison to the structure with the E123IR deleted shown in Figure 4d. **c**, Superposition of the structure of Atg3$^{\Delta NFR}$ (this study, light blue, PDB 6OJJ) and Atg3^{FL} (cyan PDB 2DYT) shows potential rearrangement of Atg3's extreme C-terminal element between E123IR-bound and -displaced forms of Atg3. Residues not visible in the prior structure are highlighted with spheres for alpha carbons. The conformation of Atg3's extreme C-terminal residues observed in the Atg3$^{\Delta NFR}$ structure is incompatible with E123IR binding due to clashing, and is consistent with NMR chemical shift perturbations of this region detected by NMR in Figure 4.

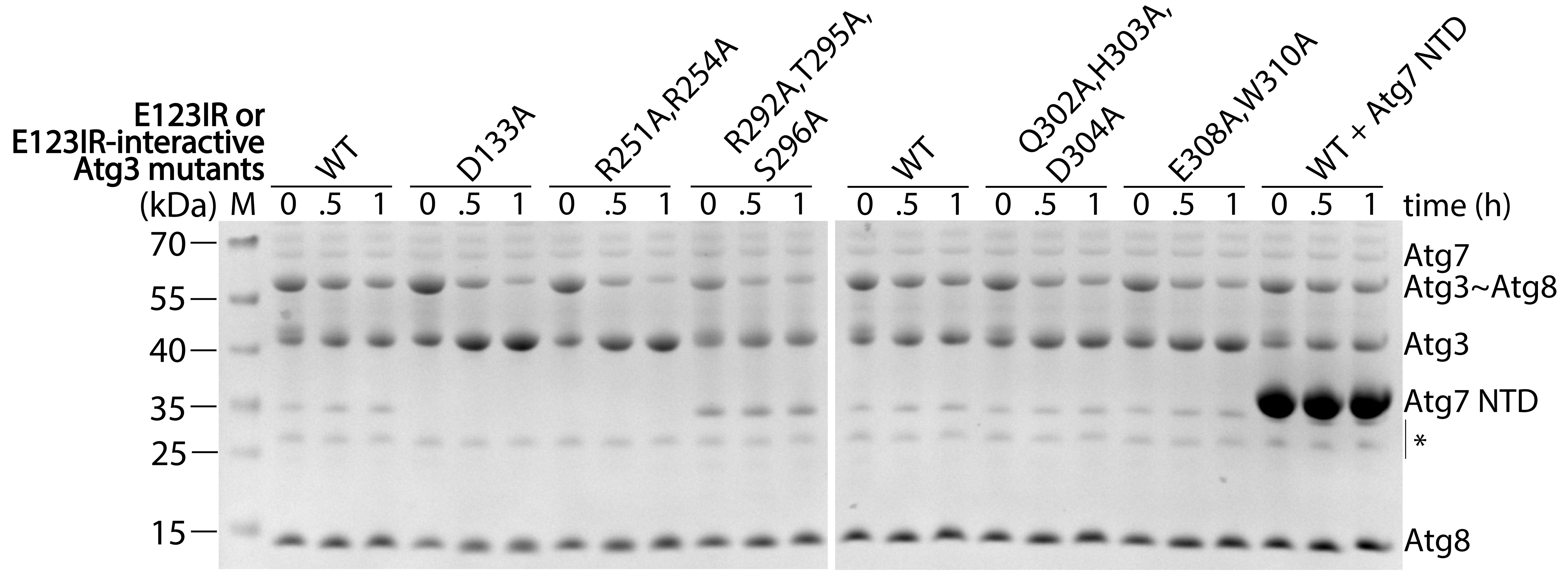

Supplementary Figure 5: Mutations in interface between Atg3's E123IR element and catalytic domain activate the Atg3~Atg8 intermediate.
Representative gel image of assay from Figure 6c.

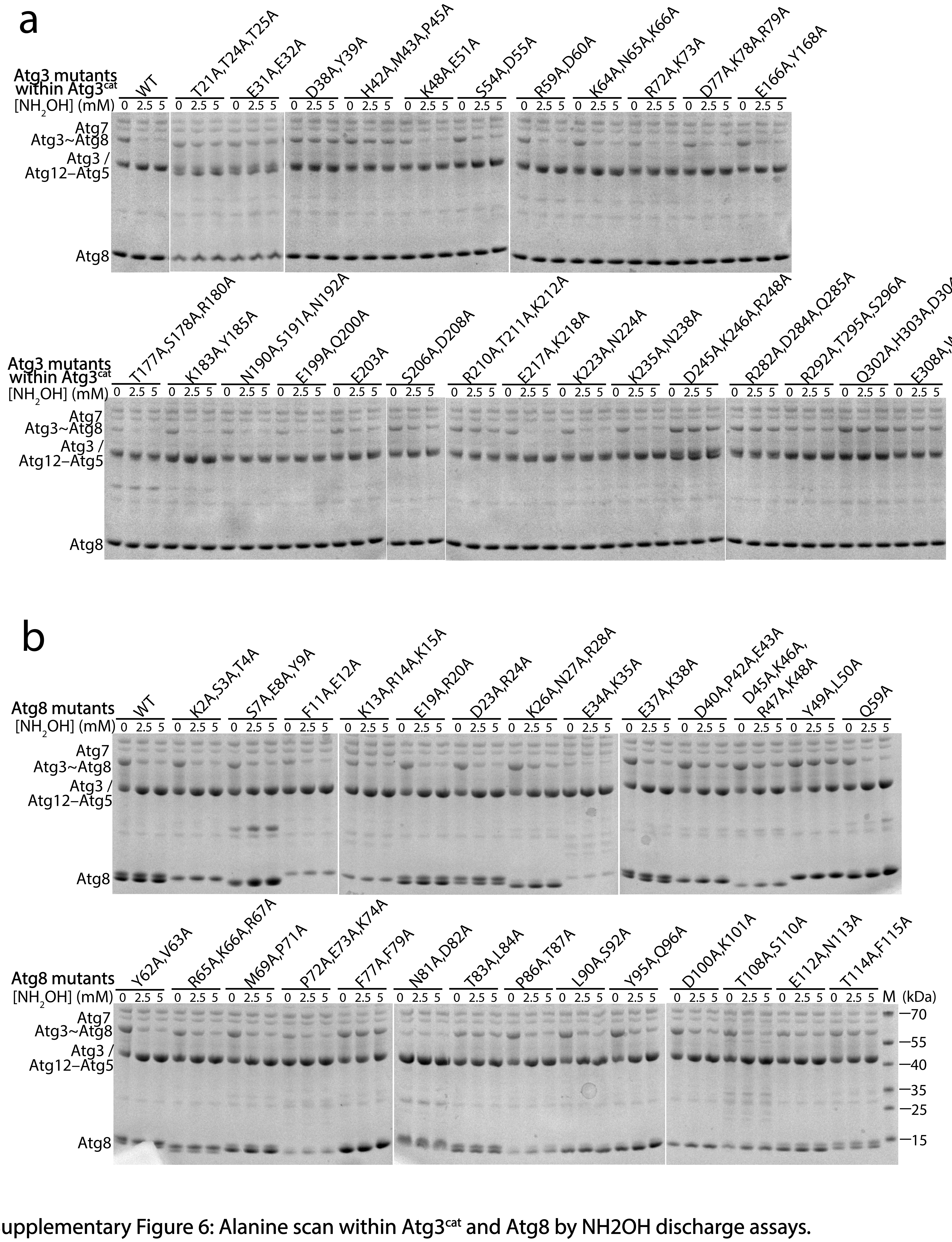

Supplementary Figure 6: Alanine scan within Atg3[cat] and Atg8 by NH2OH discharge assays.
a, Representative gel image of assay from Figure 7a. b, Representative gel image of assay from Figure 7b.

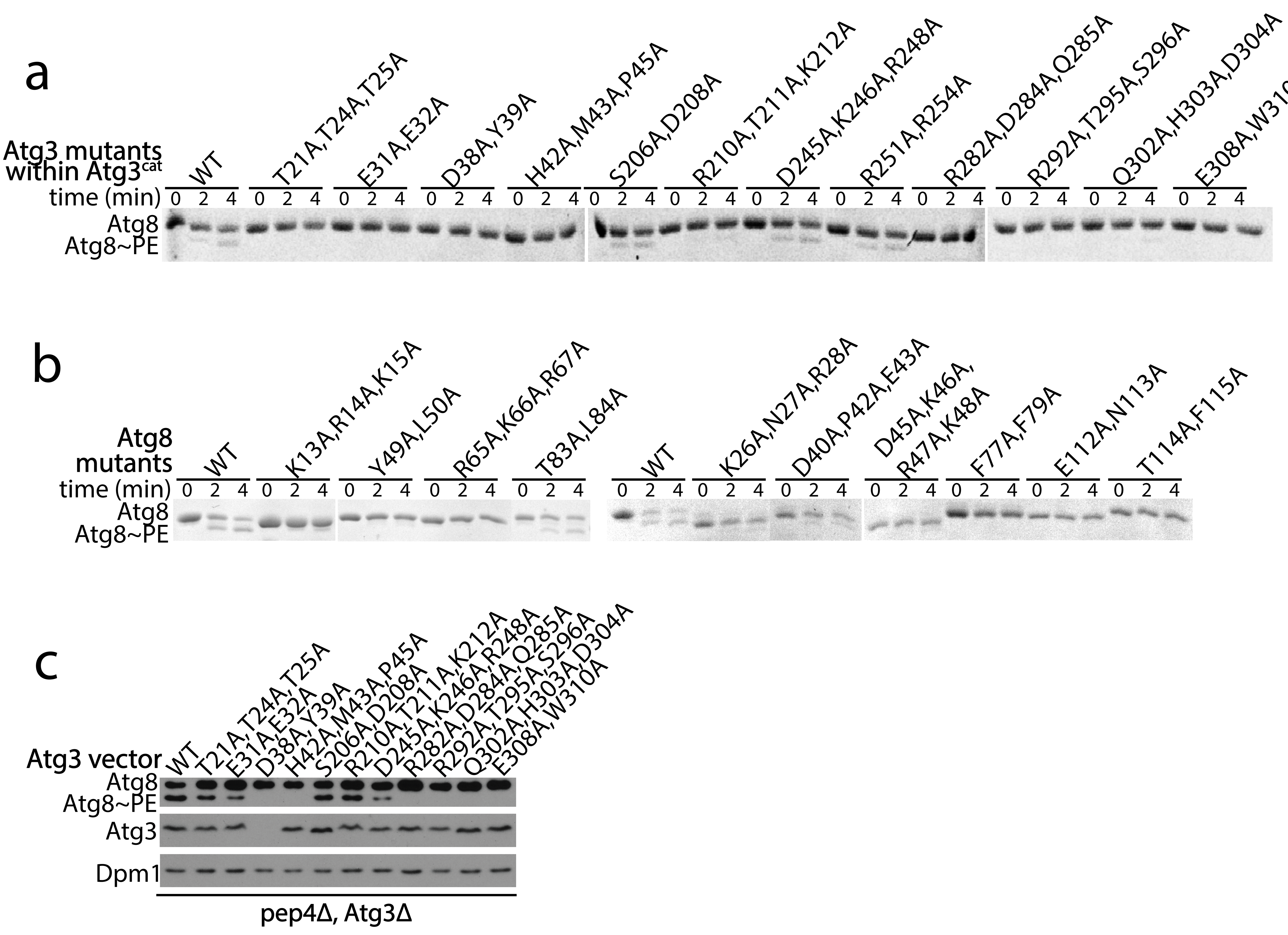

Supplementary Figure 7: Alanine mutants within $Atg3^{cat}$ and Atg8 examined by Atg8-lipidation assays in vitro and in vivo.

a, Effects of indicated mutants defective in a on E3-dependent Atg8 lipidation in vitro, in reactions with Atg7, Atg12–Atg5-Atg16, and liposomes generated from E. coli polar lipids as a source PE, and detected by migration of Atg8 in Coommassie-stained SDS-PAGE gel.

b, Effects of indicated mutants defective in b on E3-dependent Atg8 lipidation in vitro, in reactions with Atg7, Atg12–Atg5-Atg16, and liposomes generated from E. coli polar lipids as a source PE, and detected by migration of Atg8 in Coommassie-stained SDS-PAGE gel.

c, Effects of indicated mutants defective in a on Atg8 lipidation in vivo, as detected by western blot for Atg8 after 2 hours starvation of the XLY161 atg3Δpep4Δ strain of S. cerevisiae expressing either WT or mutant HA-tagged Atg3. Dpm1 is loading control.

Supplementary Table 1: Data collection and refinement statistics

	**Atg3$^{\Delta NFR}$** (ScAtg3 Δ1-18, Δ86-159)
**Data collection**	
Space group	C 1 2 1
Cell dimensions	
a, b, c (Å)	92.078, 44.557, 66.2
α, β, γ (°)	90, 102.977, 90
Resolution (Å)	30-2.40 (2.44-2.40)*
R_{merge}	0.050 (0.766)
$I / \sigma (I)$	7.9 (1.1)
$CC_{1/2}$	0.996 (0.587)
Completeness (%)	92.6 (77.3)
Redundancy	3.4 (2.8)
**Refinement**	
Resolution (Å)	26.41-2.50
No. reflections	9090
R_{work} / R_{free}	0.2127 / 0.2427
No. atoms	1519
Protein	1458
Water	61
B factors (Å^2)	51.20
Protein	51.24
Water	50.23
R.m.s. deviations	
Bond lengths (Å)	0.006
Bond angles (°)	0.918

* Values in parentheses are for highest-resolution shell.
