# Supplementary figures and images for: A switch element in the autophagy E2 Atg3 mediates allosteric regulation across the lipidation cascade
Source: Nat Commun. 2019 Aug 9;10:3600. doi: 10.1038/s41467-019-11435-y (PMC6689050; doi:10.1038/s41467-019-11435-y)

Fig. 1b

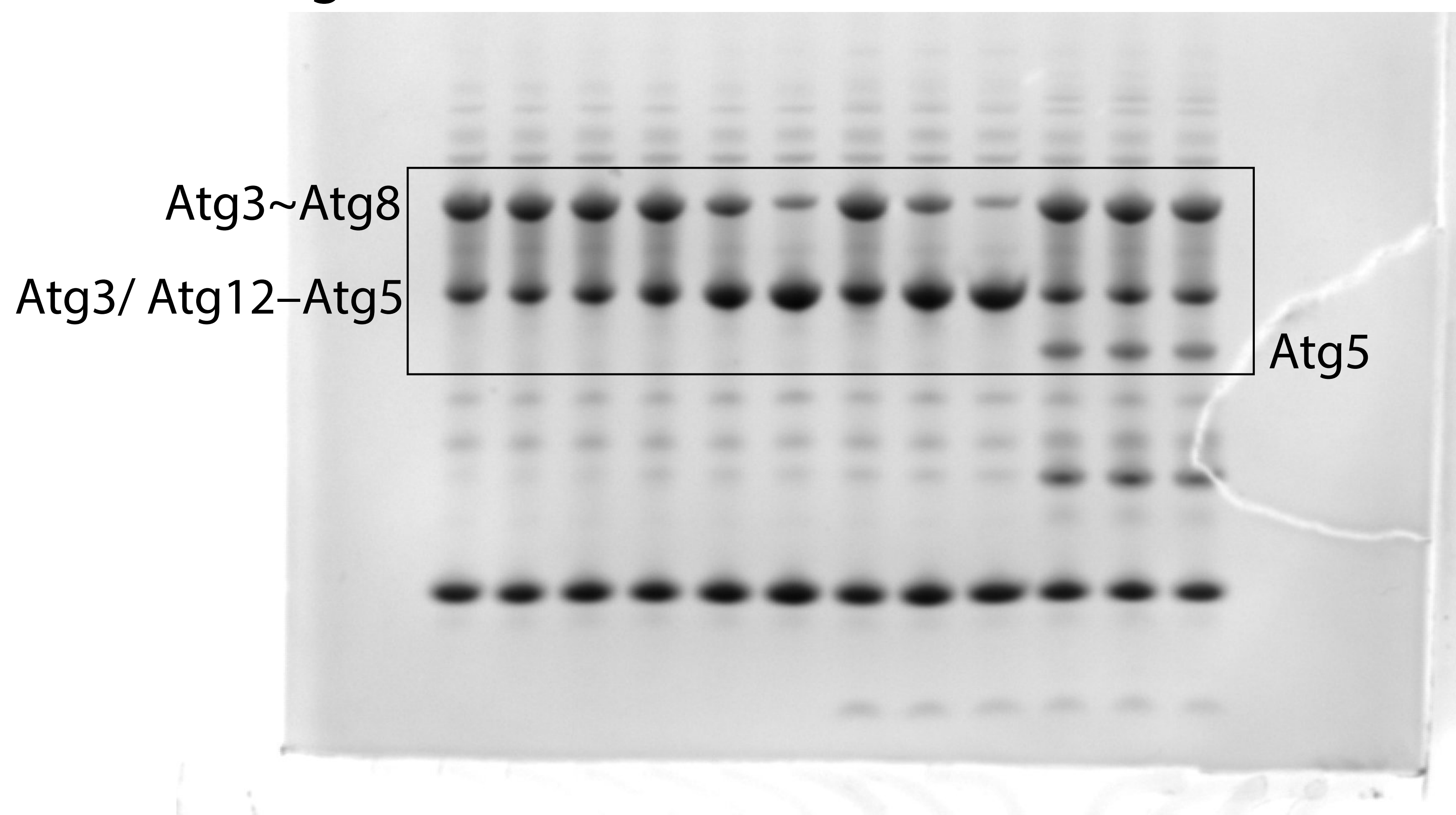

Fig. 2b

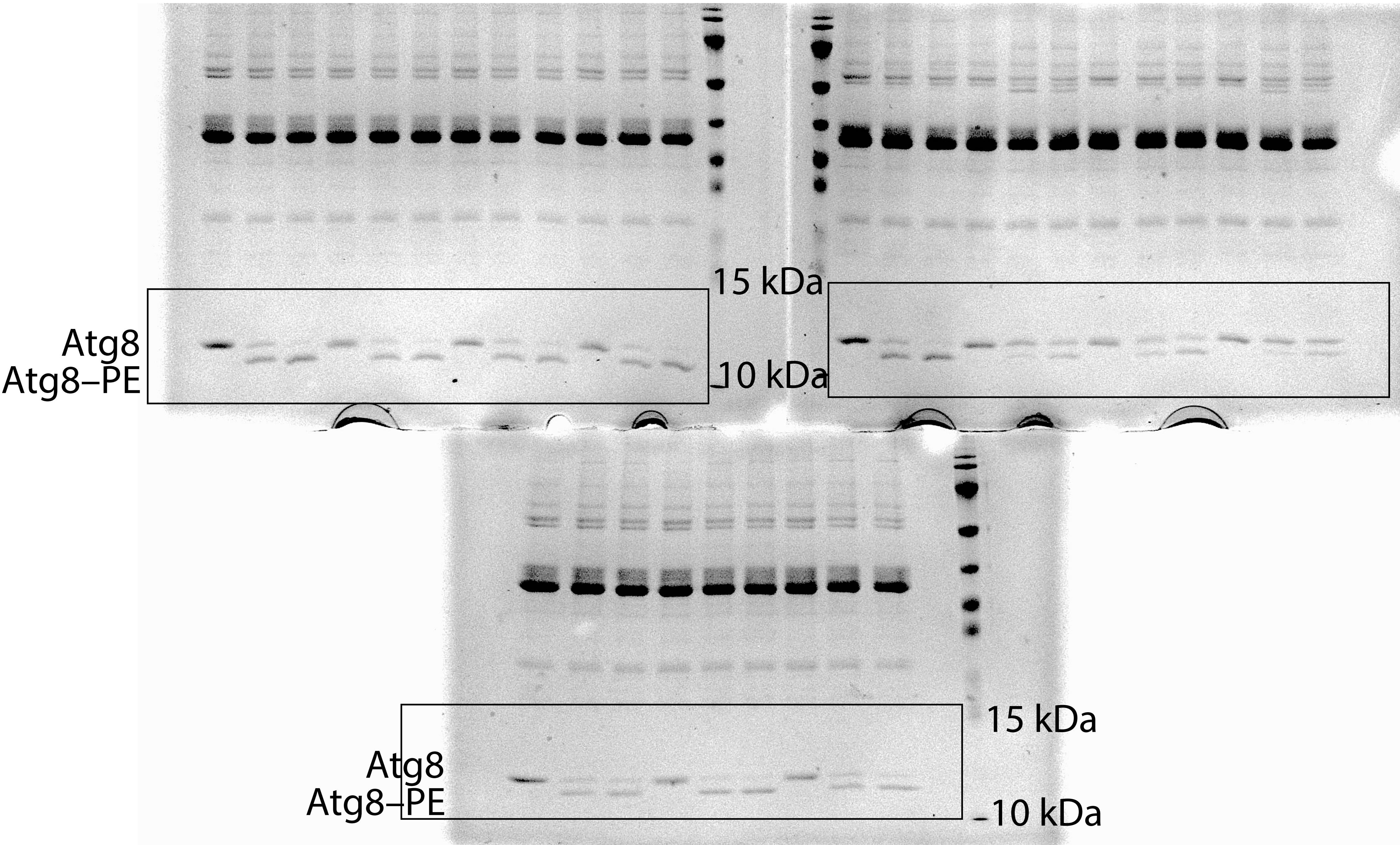

Fig. 2c

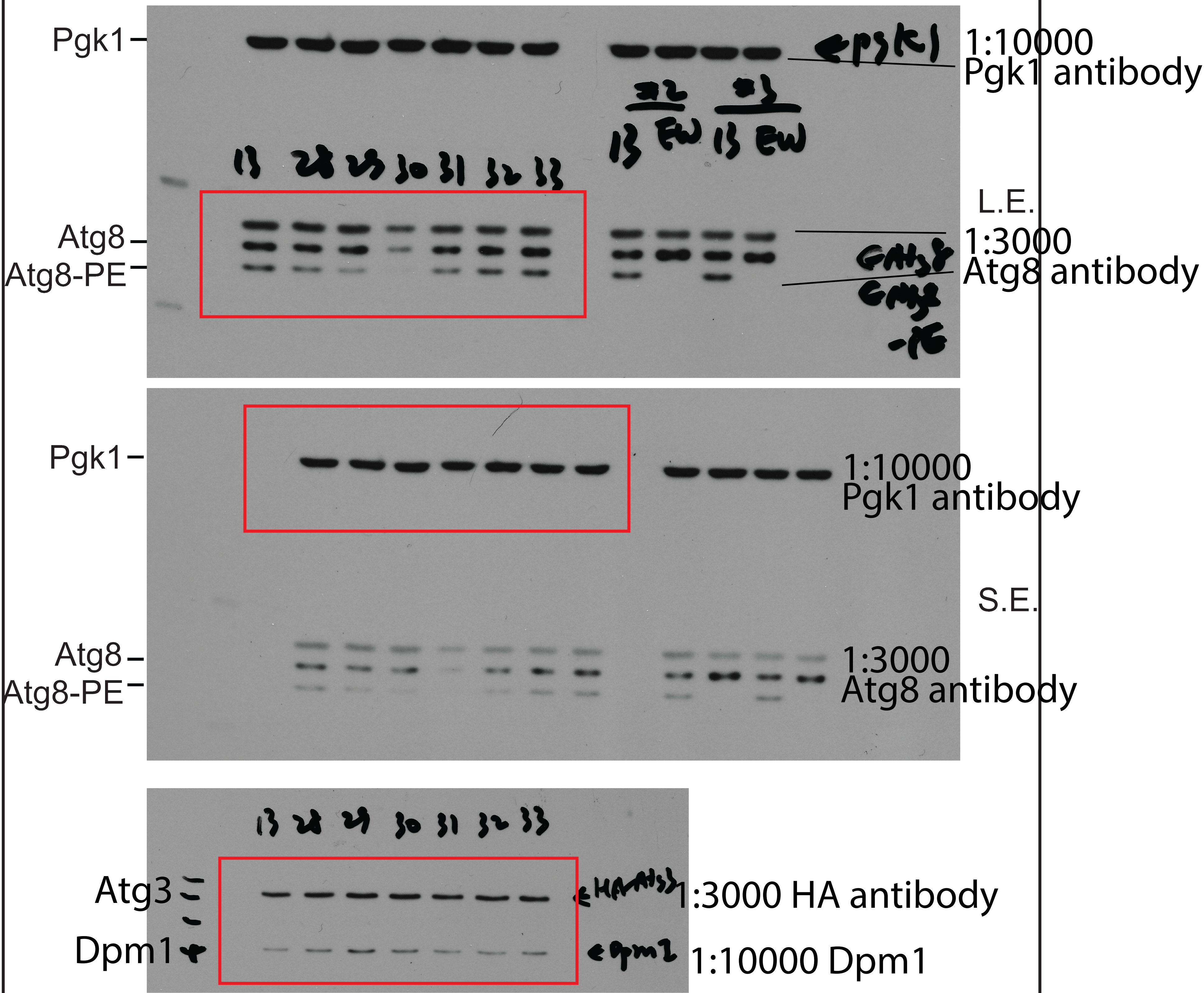

Fig. 4b

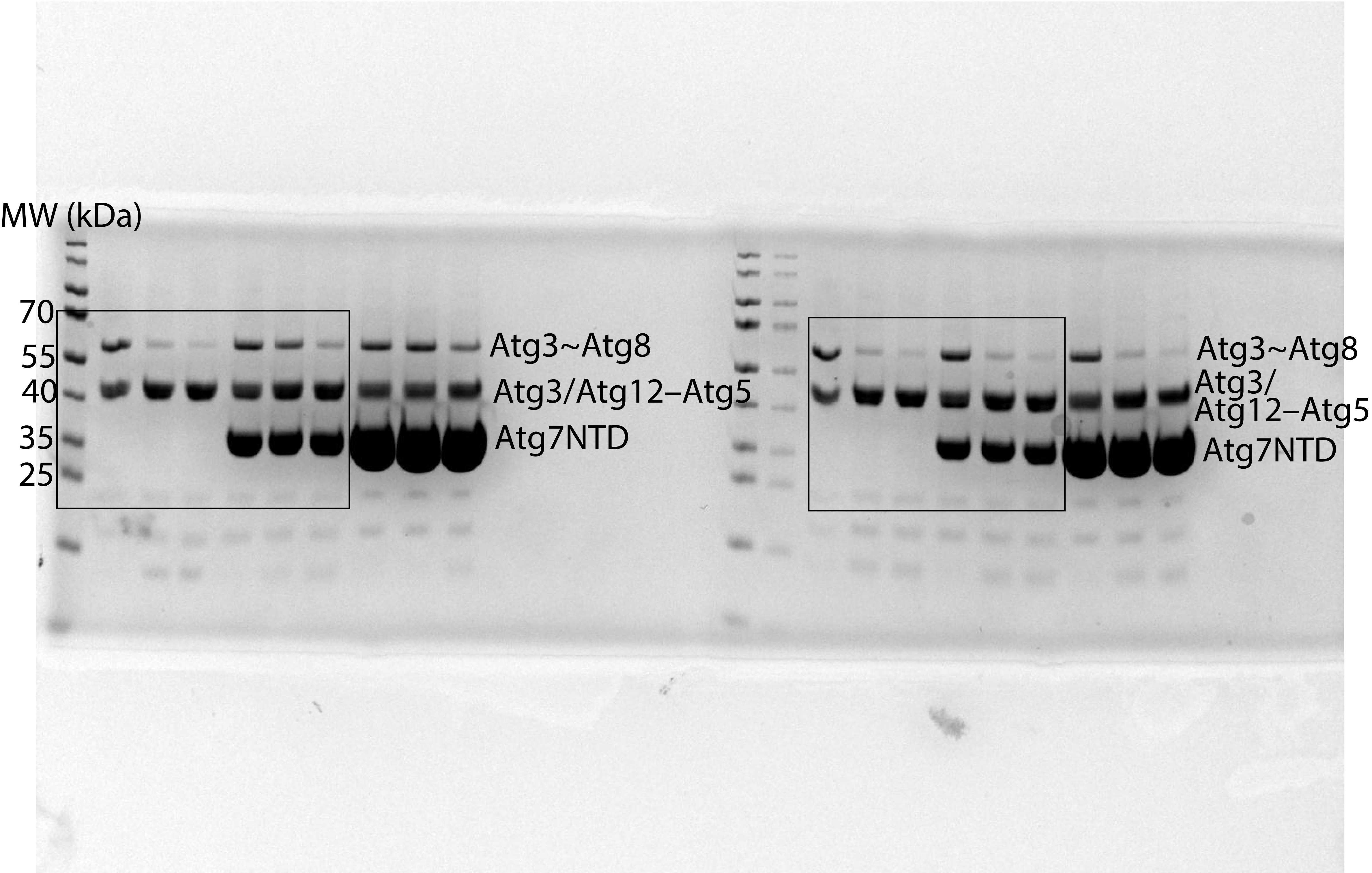

SFig. 1b

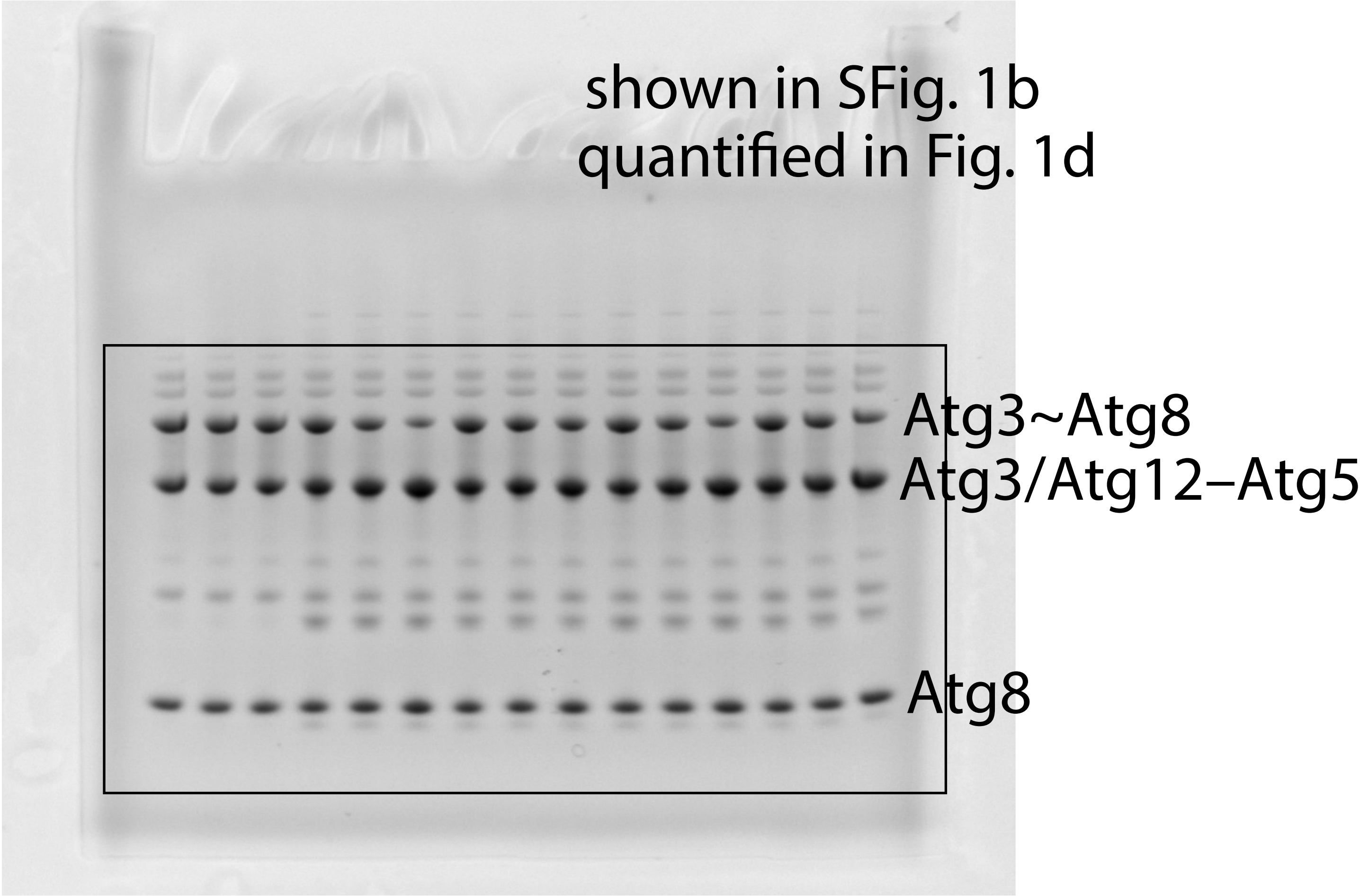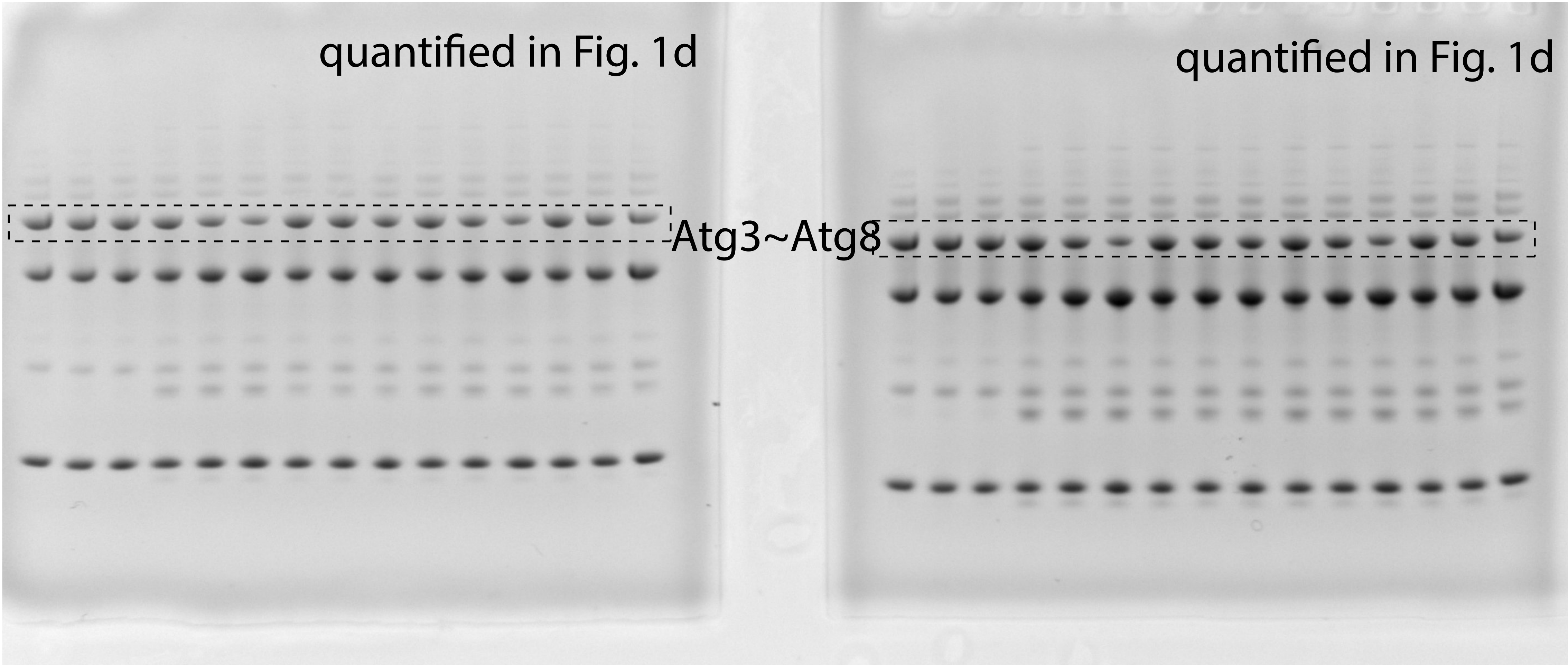

SFig. 1c

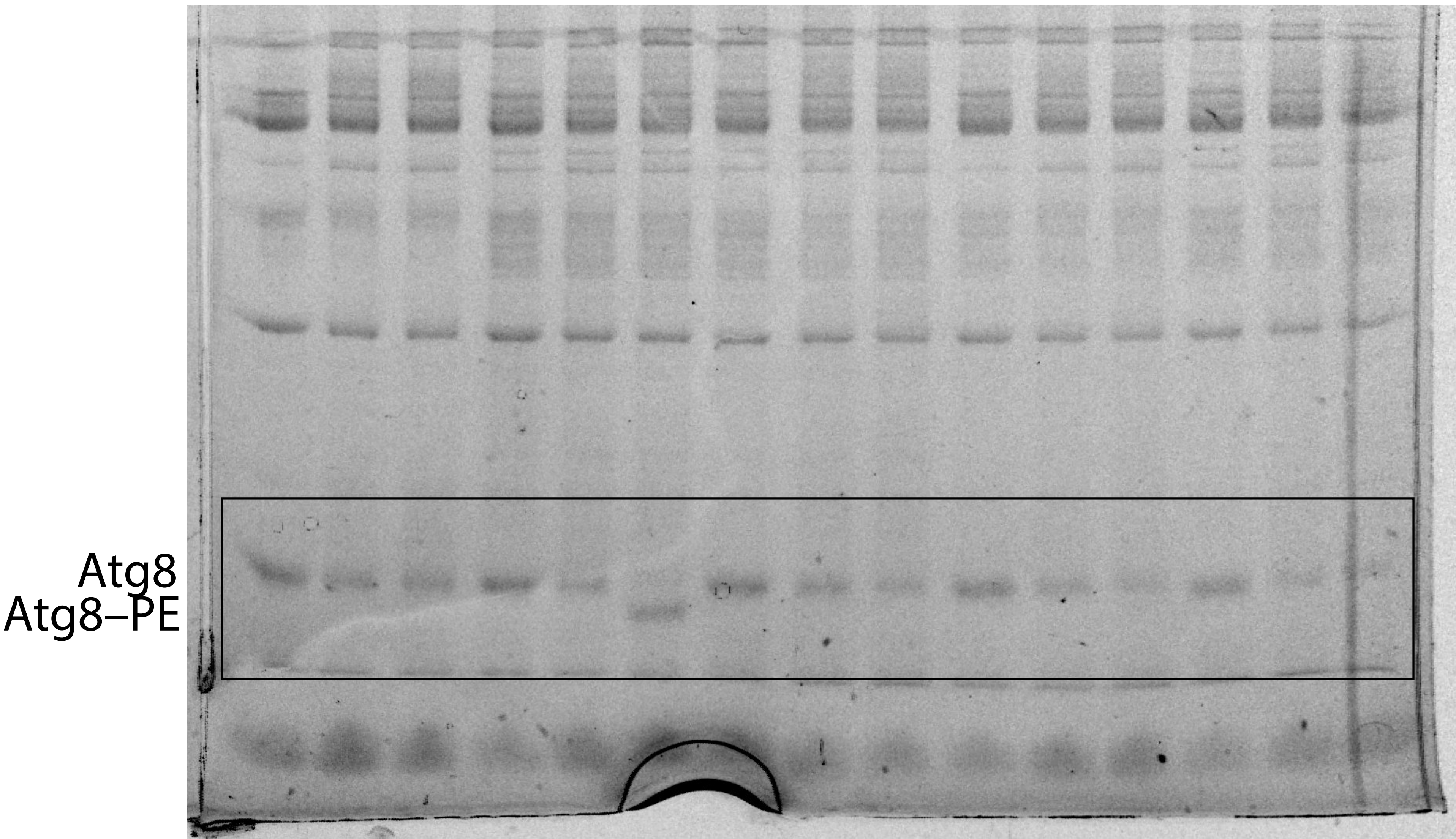

Fig. 6d

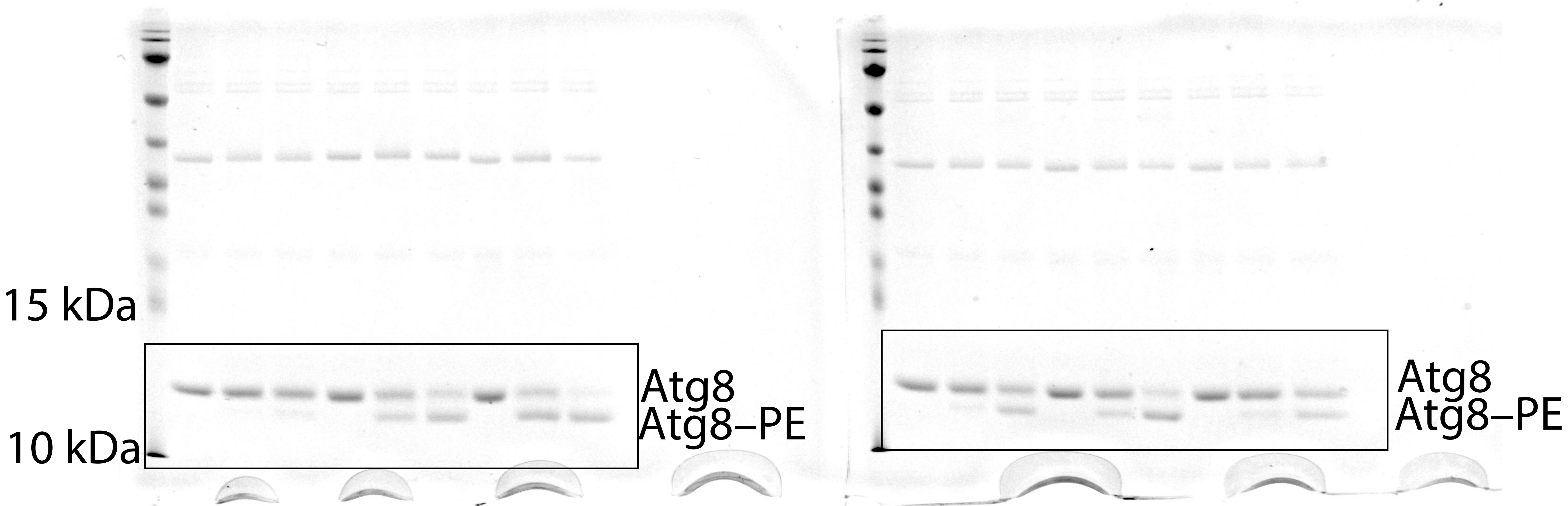

Fig. 6e

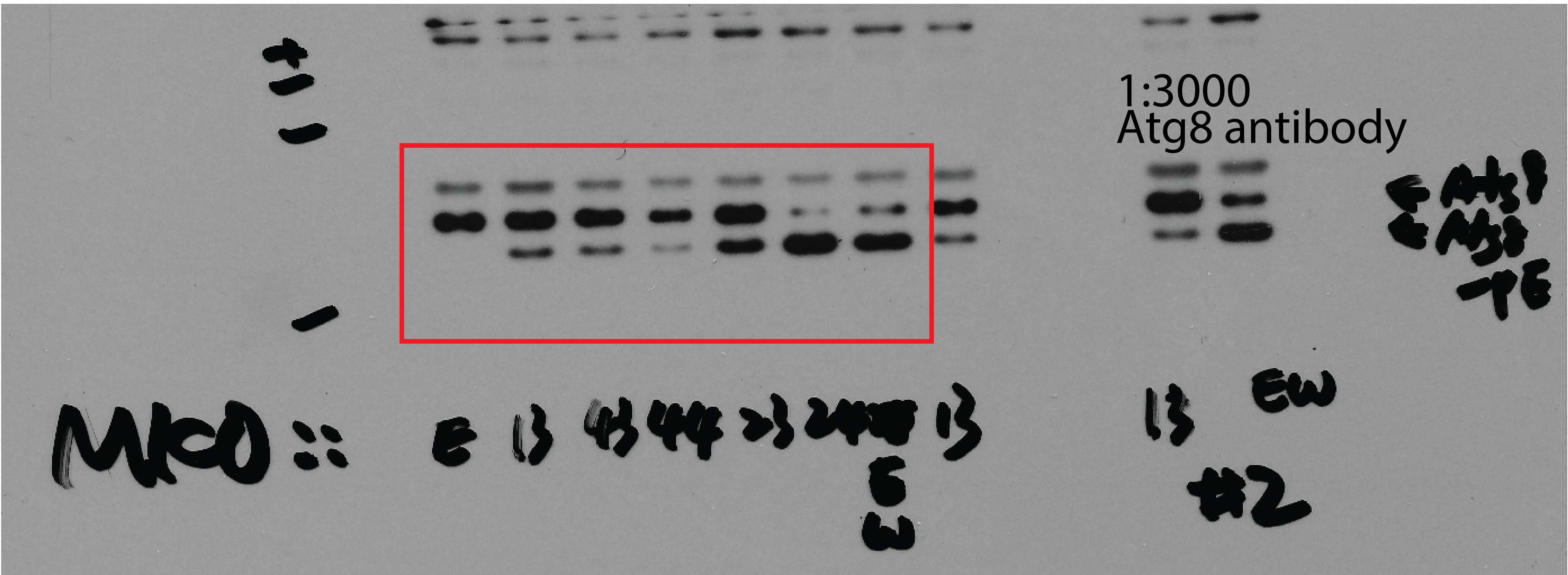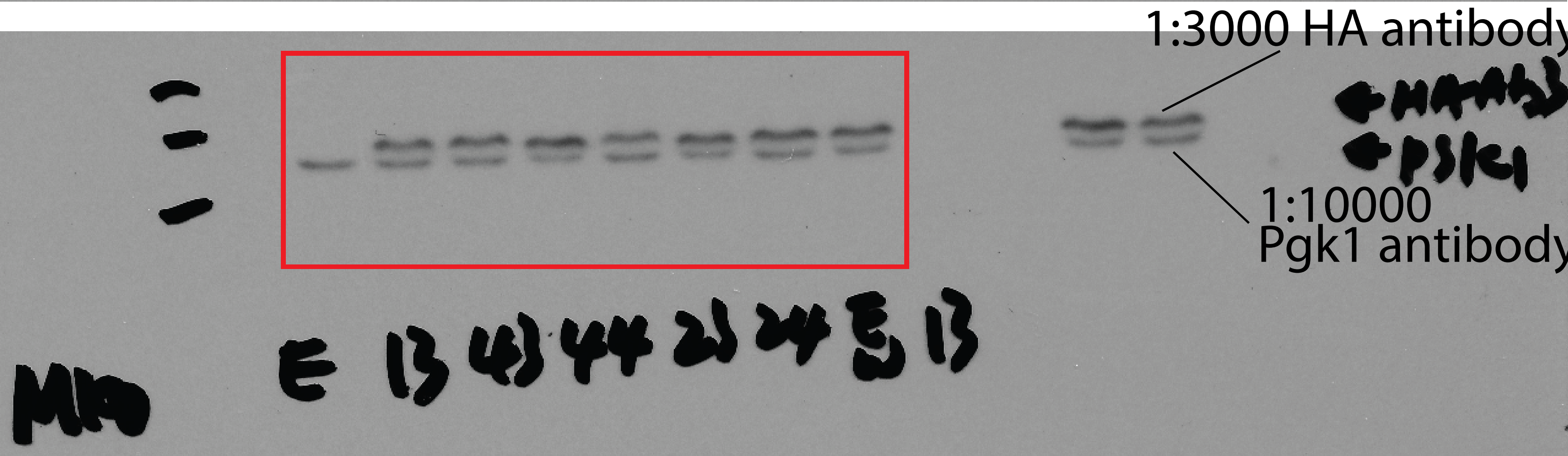

SFig. 2

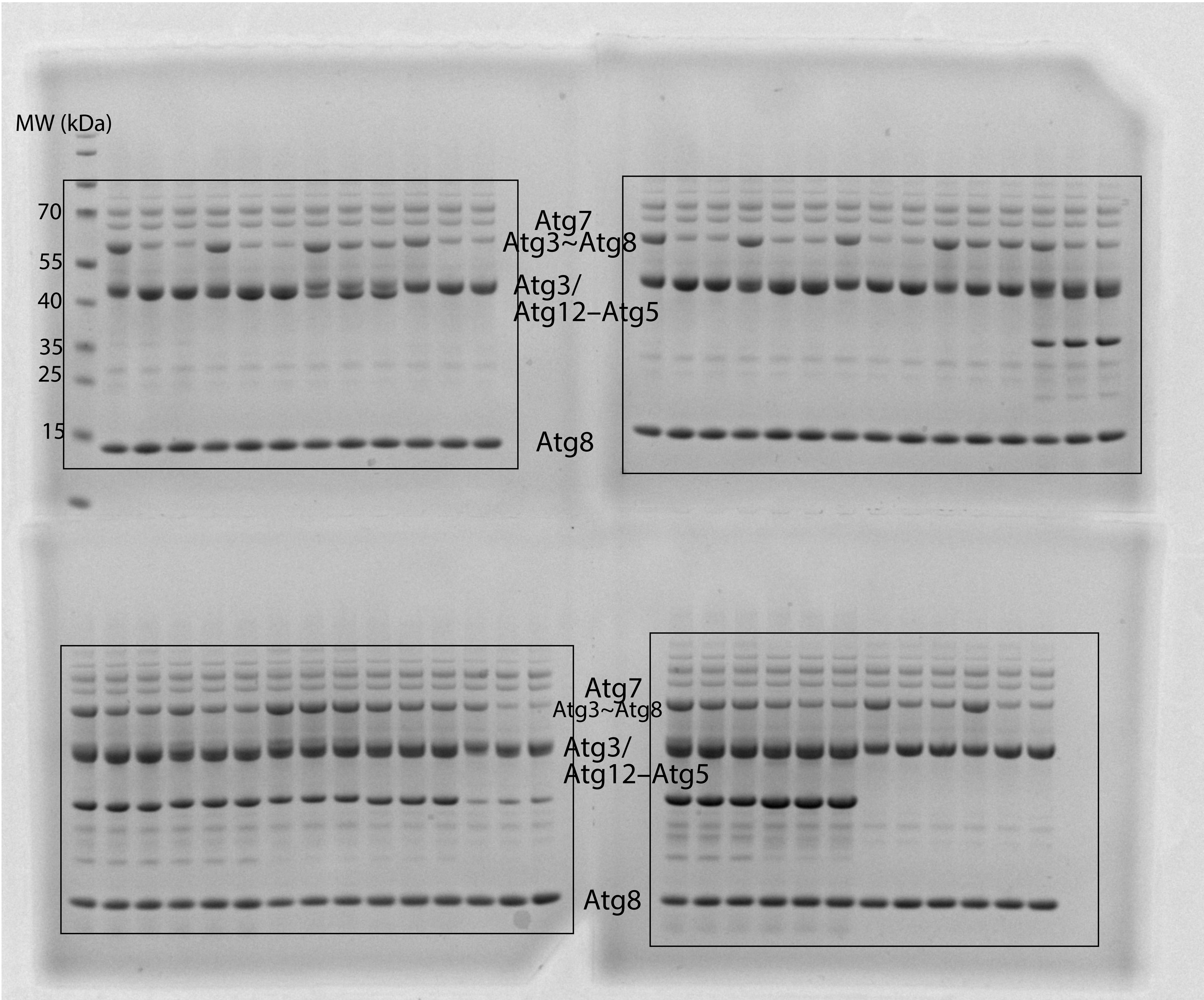

SFig. 5

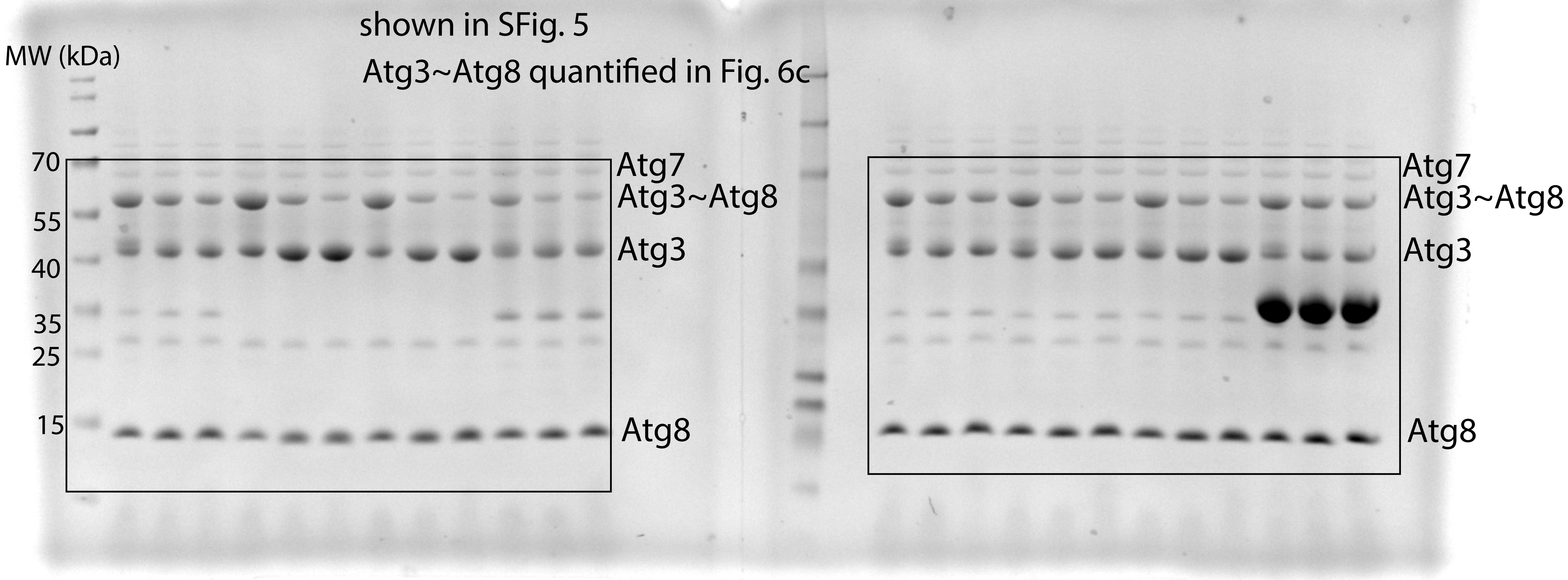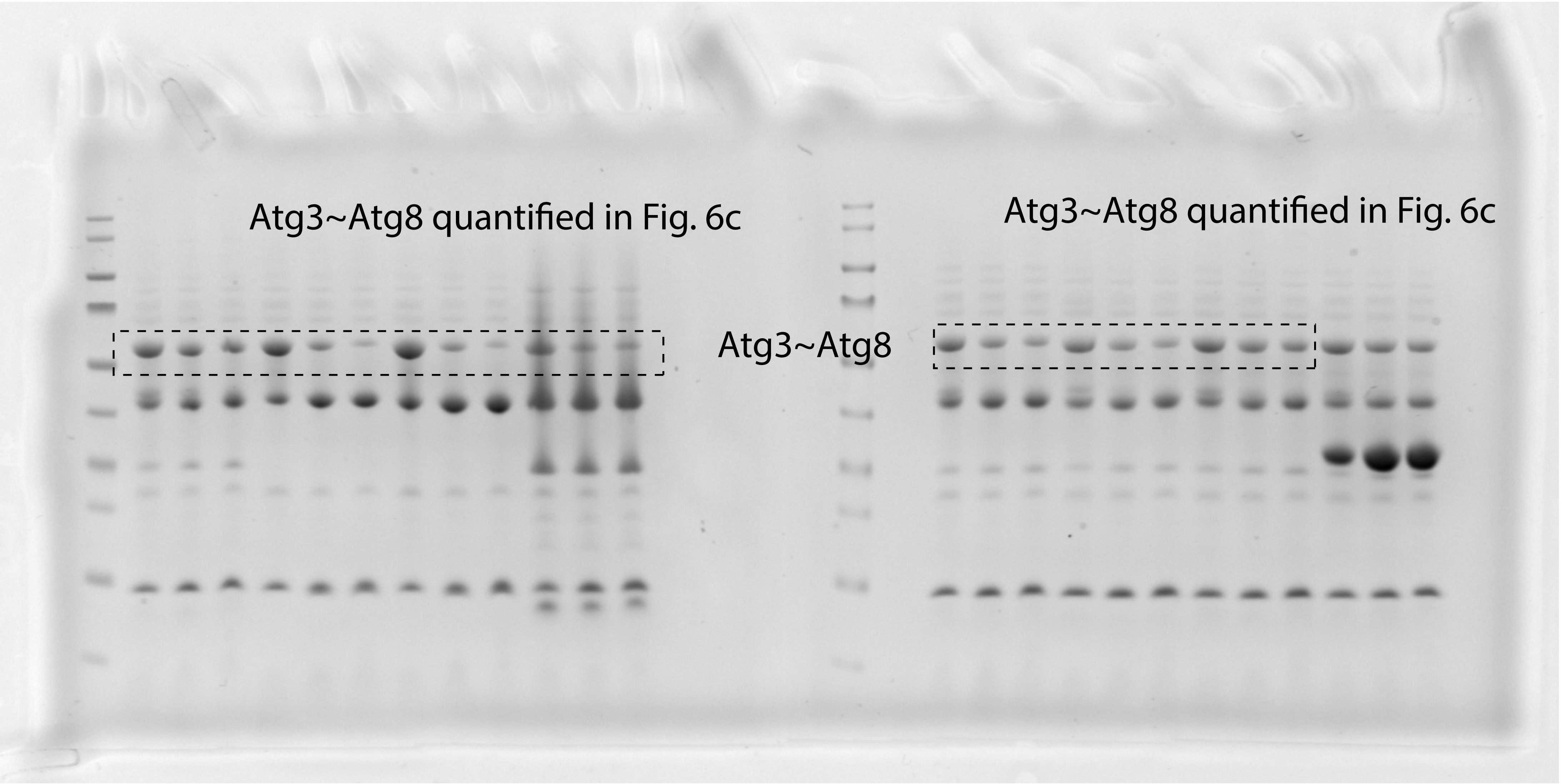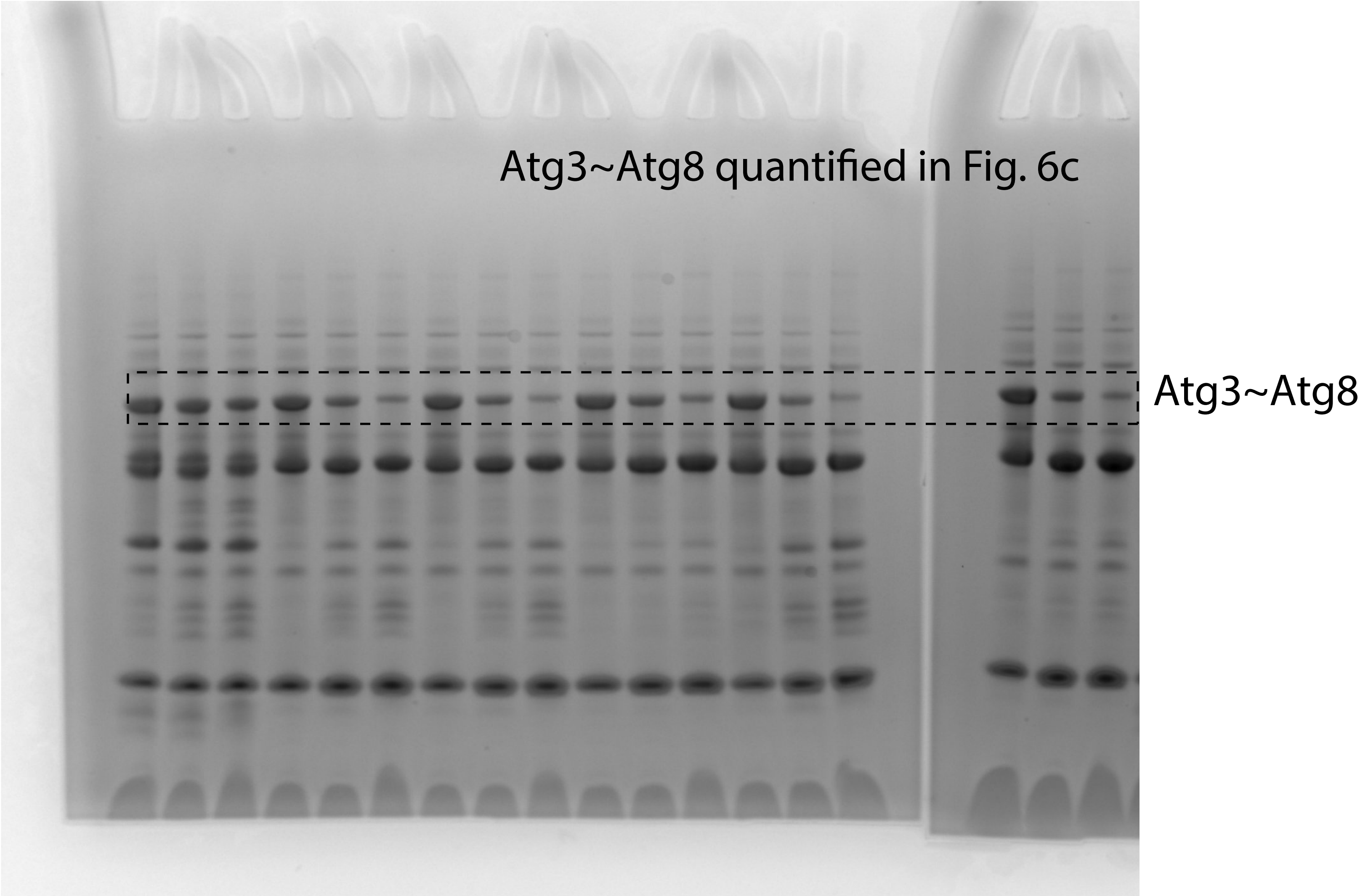

SFig.6a

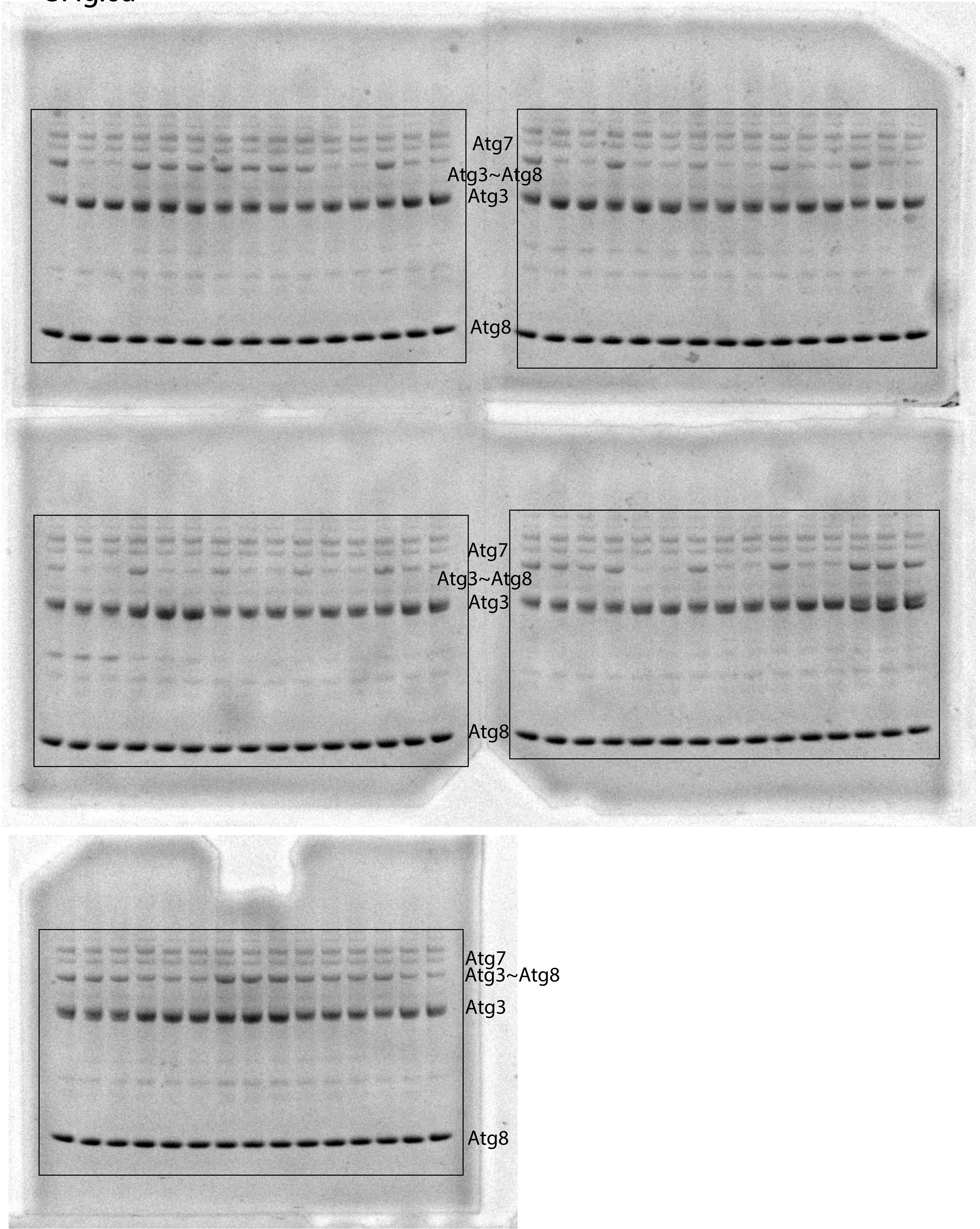

SFig. 6b

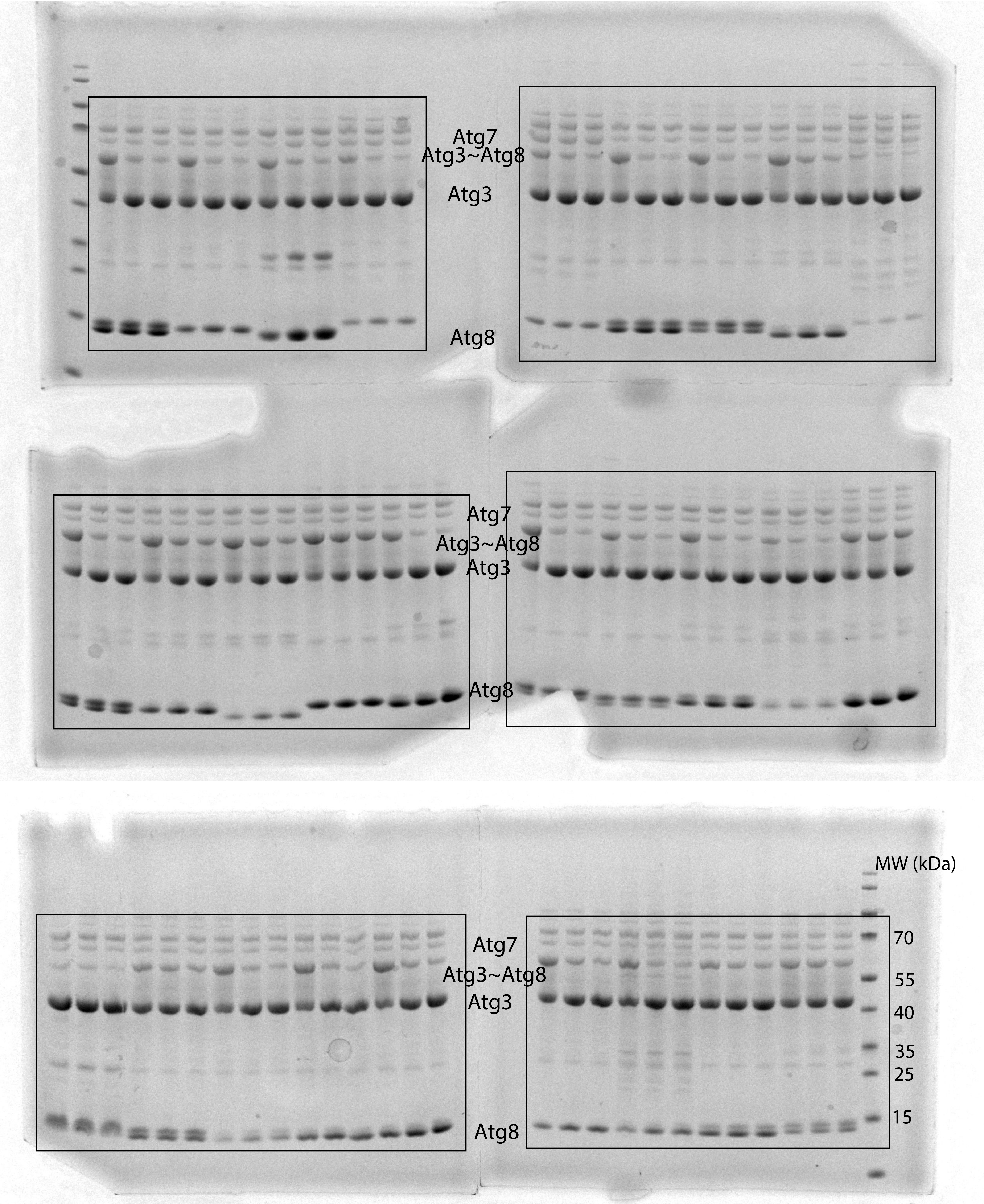

SFig. 7a

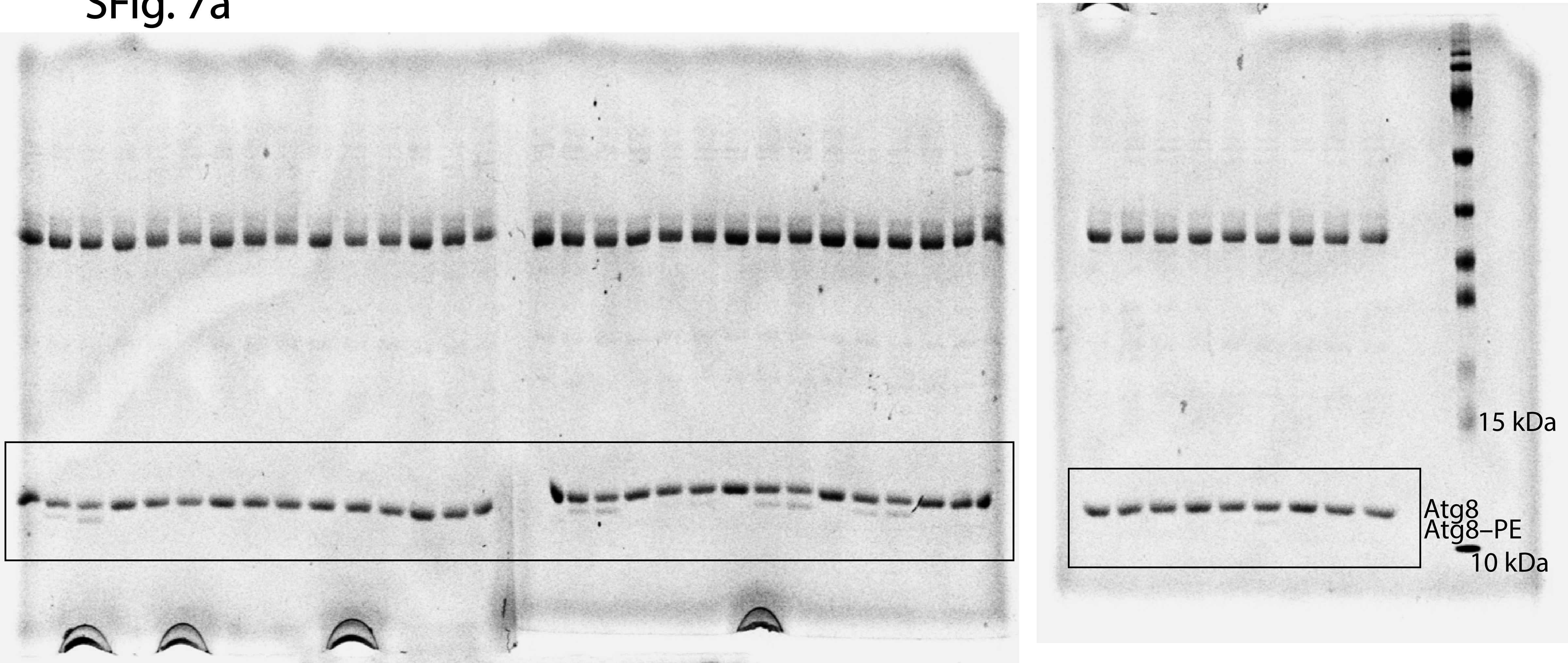

SFig. 7c

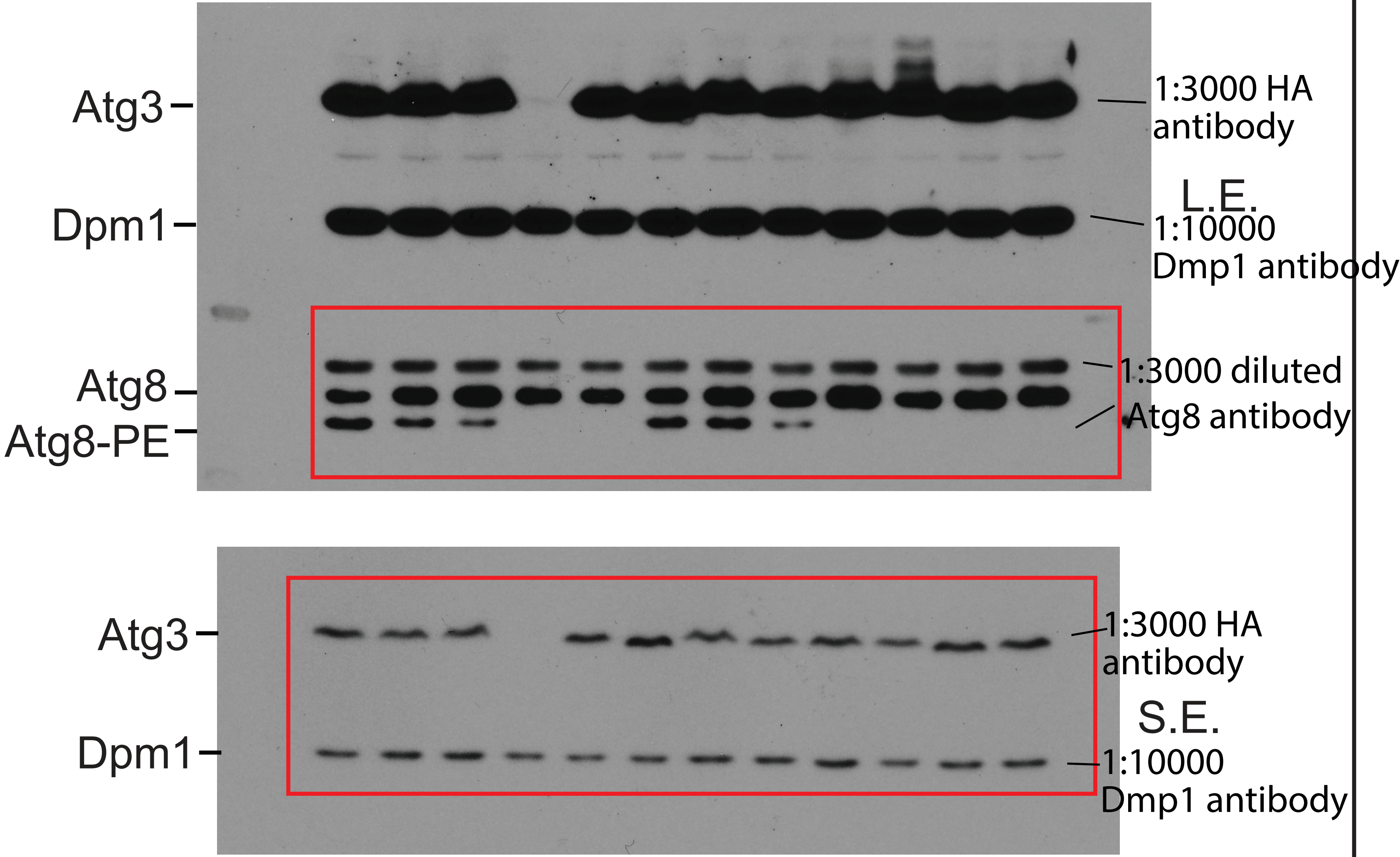

**SFig. 7b**

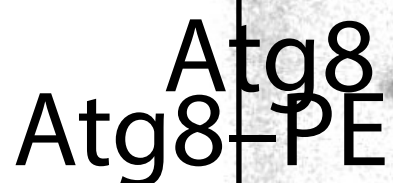

Supplement: Supplementary file 4 — Source Data [file 41467_2019_11435_MOESM4_ESM.pdf]
