# Supplementary material for: A switch element in the autophagy E2 Atg3 mediates allosteric regulation across the lipidation cascade
Source: Nat Commun. 2019 Aug 9;10:3600. doi: 10.1038/s41467-019-11435-y (PMC6689050; doi:10.1038/s41467-019-11435-y)
Supplement: Supplementary file 2 — Peer Review [file 41467_2019_11435_MOESM2_ESM.pdf]

Reviewers' comments:

Reviewer #1 (Remarks to the Author):

Zheng et al. reveal a new allosteric regulatory region in the autophagy E2 enzyme Atg3 that restrains its catalytic activity with relief by the E1 and E3 enzymes. This region was previously shown to interact with the autophagy E1 Atg7. By using NMR and biochemical assays, the authors demonstrate in this work that the region physically and functionally interacts with its own catalytic domain as well as E3 Atg12-Atg5 and therefore name the region E123IR (E1, E2, and E3 interacting region). The authors propose E123IR is able to allosterically position the catalytic cysteine in a configuration less favorable for catalysis, based on comparison of their new structure of the Atg3 catalytic domain and a published structure with the E123IR included. There are some aspects to the work that could use strengthening, but the results overall are convincing and of high impact. Suggestions are listed below.

A key aspect of the study is the structure shown in Figure 1a, 3e, 5b, which demonstrates the mechanism of interaction between the catalytic domain and E123IR. An apparent conflict however is this structure with the data shown in Figure 3c where residues 303-310 seem to have the greatest CSP with addition of Atg3-FR. In 3e/5b these residues do not seem to be closest to the E123IR. Also, mutations of these amino acids in fig. 3c seems to have a relatively mild effect in fig 5c compared to the D133A mutation. The authors should try to investigate the discrepancy of the NMR data further. For example, 303-310 appear to be in a loop region; is it possible that the CSP data is picking up a structural rearrangement with this loop? Since the bound state appears to be observable this possibility or the alternative of a closer interaction than shown in 3e/5b could be tested directly by NMR.

In addition, with the chemical shift assignments, it is possible to plot the chemical shift index for Ca and carbonyl atoms to determine whether the E123IR construct is helical, as displayed in the structure. This analysis would test further the proposed model.

It would be informative to measure binding affinity for the various interactions. One would expect relatively strong affinity for the E123IR and Atg12-Atg5 complex, based on the presented data.

The model of E123IR/cat interaction (Figure 3a) could be further interrogated by testing for binding with an I136 mutation, using the NMR approach of Figure 3d or preferably by measuring effect on binding affinity. A compensatory mutation in the catalytic domain would provide even stronger evidence for the model.

The authors should probably address in the text whether the structural change observed in Figure 4 could arise simply from the deletion itself independent of interaction. This is a more minor concern but maybe worth considering.

Very minor points
p. 3: ‘Fig. 1b’ should be ‘Fig. 1a’
Fig 3e: would be clearer with E123IR and cat domain colored differently for shifted residues

I would like to note that the Discussion section was very well done; thoughtful and a pleasure to read.

Reviewer #2 (Remarks to the Author):

This is an exceptionally strong and insightful manuscript, which gets at a big question: how does the E3 of autophagic Atg8 lipidation work? This E3, consisting of Atg5, 12, and 16, lacks homology to

ubiquitin and ubiquitin-like E3s. The answer seems to be that it allosterically activates Atg3 through the "E123IR" described here in great detail and rigor. The manuscript is appropriate for rapid publication after a few very minor corrections.

1. Typo: page 3 line 75, Fig.1b should be Fig.1a.
2. Please provide an omit electron density map of the catalytic site region in the Atg3delete-FRcrystal structure.
3. Figure labeling does not match text: in Fig.3c, is this the titration of Atg3E123IR (indicated in line 173) or AtgFR (indicated in figure) to 15N Atg3cat?
4. Personally I find the term "E123IR" too long and cumbersome. How about "AR" or "RR" for allosteric region or regulatory region?

Reviewer comments and point-by-point responses in blue:

General response to both reviewers: We are very pleased by the extremely enthusiastic responses from Reviewers! It is really nice to receive such supportive comments about our work. Thank you! Thank you also for helpful suggestions for improving our presentation. We tried to address all your suggestions, both by adding additional experiments and revising the text and figures. To assist the Reviewers, we have highlighted in yellow the portions of the main text that address comments.

Reviewer #1:

Zheng et al. reveal a new allosteric regulatory region in the autophagy E2 enzyme Atg3 that restrains its catalytic activity with relief by the E1 and E3 enzymes. This region was previously shown to interact with the autophagy E1 Atg7. By using NMR and biochemical assays, the authors demonstrate in this work that the region physically and functionally interacts with its own catalytic domain as well as E3 Atg12-Atg5 and therefore name the region E123IR (E1, E2, and E3 interacting region). The authors propose E123IR is able to allosterically position the catalytic cysteine in a configuration less favorable for catalysis, based on comparison of their new structure of the Atg3 catalytic domain and a published structure with the E123IR included. There are some aspects to the work that could use strengthening, but the results overall are convincing and of high impact.

We thank the reviewer for such kind comments! We are very pleased by the enthusiastic response!

Suggestions are listed below.

A key aspect of the study is the structure shown in Figure 1a, 3e, 5b, which demonstrates the mechanism of interaction between the catalytic domain and E123IR. An apparent conflict however is this structure with the data shown in Figure 3c where residues 303-310 seem to have the greatest CSP with addition of Atg3-FR. In 3e/5b these residues do not seem to be closest to the E123IR. Also, mutations of these amino acids in fig. 3c seems to have a relatively mild effect in fig 5c compared to the D133A mutation. The authors should try to investigate the discrepancy of the NMR data further. For example, 303-310 appear to be in a loop region; is it possible that the CSP data is picking up a structural rearrangement with this loop? Since the bound state appears to be observable this possibility or the alternative of a closer interaction than shown in 3e/5b could be tested directly by NMR.

We apologize for lack of clarity on this issue in our original manuscript. To address this in the revision, we present additional structural analyses (Supplementary Fig. 4b) and changed the text (page 6 line 37). Most important to clarify, the majority of these residues (303-308) are visible in our crystal structure of Atg3$^{\Delta NFR}$ structure, which shows a conformational change from the prior structure of full-length/E123IR-bound Atg3 (PDB code 2DYT). Superposition of the two structures shows that residues 307-308 in the conformation in the crystals of Atg3$^{\Delta NFR}$ would clash with E123IR binding, yet their nearby location suggests they would be poised to bind the E123IR in an alternative conformation, and be poised to contact Atg8 in our model of an Atg3~Atg8 intermediate. However, these residues were not observed in the prior structure, and thus at this point, we neither know their precise positions when Atg3's catalytic domain and E123IR interact in solution, nor in the Atg3~Atg8 intermediate. Nonetheless, the data suggest that they would adopt distinct conformations when favoring E123IR binding, versus when favoring formation of the activated Atg3~Atg8 intermediate. We presume that the balance between roles in these intermediates, and in charging by E1, would account for the different magnitudes of mutational effects for the different regions of Atg3 in the different assays.

In addition, with the chemical shift assignments, it is possible to plot the chemical shift index for Ca and carbonyl atoms to determine whether the E123IR construct is helical, as displayed in the structure. This analysis would test further the proposed model.

To address this, we plotted the $\Delta\delta C^{\alpha} - \Delta\delta C^{\beta}$ secondary chemical shifts of the $Atg3^{FR}$ peptide in its free form, which is the only form for which we have assignments, in Supplementary Fig. 3c in the revised manuscript. While the results show this is not a helix in isolation, it seems like intrinsic conformational plasticity could enable switching between E1, E2, and E3-bound states. This is now described in the main text on page 6 line 15.

It would be informative to measure binding affinity for the various interactions. One would expect relatively strong affinity for the E123IR and Atg12-Atg5 complex, based on the presented data.

To address this, we carefully measured the affinities of $Atg3^{FR}$ binding to $Atg3^{\Delta FR}$ and to Atg12-Atg5 respectively by monitoring effects of titrations using NMR. As predicted by the reviewer, the data indeed show that E123IR binds with higher affinity to Atg12-Atg5 than to $Atg3^{\Delta FR}$. This is now described in the revised manuscript in Fig. 2e and 3e, and in the main text at page 6 line 13.

Also, several residues in the E123IR region disappeared when titrated with Atg12~Atg5 even at 25 µM (1:0.25) concentration, suggesting that this region is in intermediate exchange. For the titrations with $Atg3^{\Delta FR}$, all the resonances in the E123IR region showed fast exchange and hence could be observed in the spectrum. This further suggests Atg12~Atg5 binding is stronger than $Atg3^{\Delta FR}$ binding.

The model of E123IR/cat interaction (Figure 3a) could be further interrogated by testing for binding with an I136 mutation, using the NMR approach of Figure 3d or preferably by measuring effect on binding affinity. A compensatory mutation in the catalytic domain would provide even stronger evidence for the model.

To address this, we obtained an $Atg3^{FR}$ peptide mutant harboring I132D L135D I136D mutations. As expected, the NMR titration experiment showed that these mutations decreased E123IR binding to $Atg3^{\Delta FR}$. This is now described in the revised manuscript in Supplementary Figure 3b and in the main text on page 6 line 8.

The authors should probably address in the text whether the structural change observed in Figure 4 could arise simply from the deletion itself independent of interaction. This is a more minor concern but maybe worth considering.

We apologize for lack of clarity on this issue in the original manuscript. We have revised the text on page 7 line 12 describing similar rearrangement of the Atg3 catalytic center when the E123IR was dislodged in a different manner, by Atg7 binding (Kaiser et al., 2012; Yamaguchi et al., 2012), although at the time, and even in a subsequent study (Sakoh-Nakatogawa et al., 2013), a role for displacing the E123IR was not considered. Nonetheless, the prior data support the notion that the structural change does not simply arise from the deletion itself, and instead contributes to allosteric regulation within the E1-E2-E3 cascade.

Very minor points
p. 3: ‘Fig. 1b’ should be ‘Fig. 1a’

We have corrected this typo.

Fig 3e: would be clearer with E123IR and cat domain colored differently for shifted residues

We appreciate the suggestion and have changed the color (Fig. 3f) in the revised manuscript).

I would like to note that the Discussion section was very well done; thoughtful and a pleasure to read.

This is such a thoughtful comment! We worked hard on preparing the manuscript, and it is extremely gratifying to receive such encouraging feedback. Thank you!

Reviewer #2

This is an exceptionally strong and insightful manuscript, which gets at a big question: how does the E3 of autophagic Atg8 lipidation work? This E3, consisting of Atg5, 12, and 16, lacks homology to ubiquitin and ubiquitin-like E3s. The answer seems to be that it allosterically activates Atg3 through the "E123IR" described here in great detail and rigor. The manuscript is appropriate for rapid publication after a few very minor corrections.

We thank the reviewer for such kind comments! We are very pleased by the enthusiastic response!

1. Typo: page 3 line 75, Fig.1b should be Fig.1a.

We fixed the typo.

2. Please provide an omit electron density map of the catalytic site region in the Atg3delete-FRcrystal structure.

We now show the simulated annealing/omit electron density over the catalytic site region in Fig. 4c of the revised manuscript.

3. Figure labeling does not match text: in Fig.3c, is this the titration of Atg3E123IR (indicated in line 173) or AtgFR (indicated in figure) to 15N Atg3cat?

We thank the reviewer for noticing this and have made the correction.

4. Personally I find the term "E123IR" too long and cumbersome. How about "AR" or "RR" for allosteric region or regulatory region?

While we do recognize the cumbersome nature of the term E123IR, we felt that given the complexity of protein-protein interactions and presumably numerous allosteric and regulatory regions within autophagy proteins, we should keep the term “E123IR” to describe this specific element.
